# Clinical and resting-state EEG changes following neurofeedback in adult refugees with persistent trauma-related symptoms: a transdiagnostic pre–post observational study

**DOI:** 10.3389/fpsyt.2026.1954165

**Published:** 2026-10-07

**Authors:** Mirjana Askovic, Matteo Mascelloni, Sejla Murdoch, Jorge Aroche, Ed Dean Hamlin, Anthony W. F. Harris

**Affiliations:** 1 New South Wales Service for the Treatment and Rehabilitation of Torture and Trauma Survivors (STARTTS), Sydney, NSW, Sydney, Australia; 2 Specialty of Psychiatry, Sydney Medical School, University of Sydney, Sydney, NSW, Australia; 3 Brain Dynamics Centre, Westmead Institute for Medical Research, Westmead, NSW, Australia; 4 School of Electrical and Computer Engineering, Faculty of Engineering, University of Sydney, Sydney, NSW, Australia; 5 School of Psychology, Faculty of Science, University of Sydney, Sydney, NSW, Australia; 6 Institute for Applied Neuroscience, Candler, NC, United States; 7 Department of Psychiatry, School of Medicine, University of North Carolina at Chapel Hill, Chapel Hill, NC, United States

**Keywords:** alpha oscillations, chronic trauma, electroencephalography (EEG), neurofeedback, psychological interventions, refugees, resting-state EEG, transdiagnostic

## Abstract

**Background:**

Neurofeedback has shown promise as an adjunctive intervention for persistent trauma-related psychopathology, but its electrophysiological effects in refugees following war, torture, and forced displacement remain poorly understood. This study investigated changes in resting-state alpha electrophysiology during trauma treatment incorporating individualised neurofeedback and whether these changes related to clinical improvement.

**Methods:**

This retrospective pre-post observational study included 53 adult refugees with persistent trauma-related symptoms who completed resting-state electroencephalography (EEG) before and after multidisciplinary treatment incorporating neurofeedback, trauma-focused counselling and pharmacotherapy. Participants received a mean of 38.7 neurofeedback sessions after remaining symptomatic despite previous treatment. Clinical improvement was assessed retrospectively using the Clinical Global Impression–Improvement (CGI-I) scale. Resting-state EEG analyses examined changes in global alpha amplitude, alpha reactivity, peak alpha frequency (PAF), and alpha coherence. Neurofeedback protocol implementation was characterised across 2,361 protocol applications, and secondary analyses examined electrophysiological differences between responders and non-responders and associations between electrophysiological and clinical change.

**Results:**

Thirty-three participants (62.3%) demonstrated clinically meaningful improvement following multidisciplinary treatment incorporating neurofeedback. Resting-state EEG demonstrated increased global alpha amplitude (FDR-adjusted p = .018) and reduced occipital alpha reactivity (FDR-adjusted p = .013), whereas peak alpha frequency and alpha coherence did not change significantly following correction for multiple comparisons. Exploratory analyses suggested different patterns of eyes-open and eyes-closed alpha change in responders and non-responders; however, the difference between groups was not statistically significant (t(51) = 0.88, p = .381, d = 0.24). Changes in electrophysiological measures were not significantly associated with clinical improvement. Protocol analysis demonstrated variability in electrode placement, whereas reward frequencies remained clustered within the alpha range throughout treatment.

**Conclusions:**

Individualised neurofeedback delivered within routine multidisciplinary care was associated with clinically meaningful improvement in two-thirds of refugees with persistent trauma-related psychopathology, alongside selective changes in resting-state alpha electrophysiology. These findings extend current understanding of the electrophysiological changes observed during treatment incorporating neurofeedback in a clinically complex refugee population. Although the retrospective observational design limits conclusions regarding causality, the findings support investigation of neurofeedback as an adjunctive intervention for refugees who remain symptomatic despite previous treatment and identify resting-state alpha reactivity as a promising electrophysiological measure of treatment-associated change.

## 1 Introduction

Refugees and asylum seekers exposed to war, torture, persecution, and forced displacement experience substantially elevated rates of post-traumatic stress disorder (PTSD), depression, anxiety disorders, prolonged grief disorder, and broader complex trauma-related psychopathology compared with the general population (1–4). Meta-analyses consistently report high prevalence of psychiatric morbidity in forcibly displaced populations, with pooled prevalence estimates for PTSD and depression commonly exceeding 30% and similarly elevated rates of anxiety observed amongst populations exposed to ongoing conflict and instability (1, 3, 4). Refugee mental health difficulties are influenced not only by exposure to war-related violence, torture, bereavement, and forced migration, but also by ongoing post-migration stressors including uncertain asylum status, financial hardship, discrimination, social isolation, and disrupted access to healthcare, all of which contribute to persistent psychological distress and functional impairment that may continue for years after resettlement (5, 6).

Refugee populations exposed to chronic trauma rarely present with isolated psychiatric disorders. Instead, high rates of comorbid PTSD, depression, anxiety, dissociative symptoms, somatic symptoms, and broader complex trauma-related psychopathology have been reported across forcibly displaced populations (2, 7–9). Our previous neurofeedback study, involving a substantially overlapping cohort (10), reflected this clinical complexity. Although all participants met criteria for PTSD, many also presented with comorbid major depression, sleep–wake disorders, somatic symptoms, anxiety disorders, dissociative disorders, persistent complex bereavement, and substance-related or addictive disorders. This pattern of extensive psychiatric comorbidity is increasingly conceptualised within transdiagnostic frameworks, which view chronic trauma-related psychopathology as reflecting shared disturbances across multiple diagnostic categories and support interventions targeting common underlying regulatory processes rather than diagnosis-specific symptom clusters alone (2).

Contemporary neurobiological models conceptualise the diverse clinical manifestations of chronic trauma as arising not from dysfunction within isolated brain regions but from dysregulated interactions across distributed cortical and subcortical circuits, including the amygdala, hippocampus, insula, medial prefrontal cortex, and the salience, default mode, and executive control networks (11, 12). This altered network coordination has been associated with hypervigilance, impaired contextual processing, disrupted self-related cognition, emotional dysregulation, and reduced cognitive flexibility (11, 12). More recent models further propose that disrupted somatic sensory integration may contribute to impaired self-regulation and altered embodiment by limiting the integration of bodily experience with higher-order emotional and cognitive processes (13). At a physiological level, these alterations may involve abnormalities in oscillatory mechanisms supporting cortical inhibition, sensory gating, and the dynamic allocation of attentional resources, reducing the nervous system’s capacity to selectively suppress irrelevant internal and external information (14–16). These network- and systems-level processes are increasingly investigated using electrophysiological measures that characterise resting-state neural oscillatory activity and functional connectivity.

Electroencephalography (EEG) provides a non-invasive method for examining neural oscillations and functional network activity associated with these regulatory processes. Resting-state EEG studies in PTSD have identified abnormalities in alpha oscillations and functional connectivity, supporting the use of resting-state electrophysiological measures to investigate trauma-related alterations in large-scale neural regulation (17). Amongst the major EEG frequency bands, alpha oscillations have received particular attention because they represent the dominant rhythm during resting wakefulness and are thought to play a central role in cortical inhibition, sensory gating, attentional allocation, and large-scale neural communication (14, 15). Several characteristics of alpha oscillatory activity can be quantified using resting-state EEG, each providing information about different aspects of neural regulation. Alpha amplitude is one of the principal characteristics of alpha oscillatory activity and reflects its functional state. Reduced posterior resting-state alpha amplitude has been associated with increased sensory cortical excitability, impaired sensory gating, reduced cortical inhibitory processing, and greater hypervigilance in PTSD (18). Task-based EEG studies have also demonstrated altered alpha oscillatory dynamics during traumatic memory retrieval, suggesting that trauma-related abnormalities in alpha activity extend beyond resting-state recordings (19).

Alpha reactivity, the modulation of alpha activity between eyes-closed and eyes-open resting states, reflects state-dependent modulation of posterior alpha oscillations (20). The transition between eyes-closed and eyes-open resting states provides an index of cortical responsiveness to changing sensory demands (14, 15). Unlike static measures of resting-state alpha power, alpha reactivity reflects the brain’s capacity to dynamically modulate oscillatory activity across behavioural states and may therefore provide insight into broader aspects of neural regulatory flexibility. Although alpha reactivity itself has received relatively little attention in trauma research, emerging evidence suggests that trauma-related alterations in resting-state alpha activity may differ between eyes-open and eyes-closed conditions. Reduced temporal–posterior alpha power has been observed during eyes-open, but not eyes-closed, resting-state EEG in adolescents exposed to complex childhood trauma (21). Similarly, adults with PTSD have demonstrated reduced posterior alpha power during eyes-open resting-state EEG, a finding interpreted as reflecting diminished cortical inhibition and increased sensory hyperresponsivity associated with hypervigilance (18). Although these studies did not directly examine alpha reactivity, they suggest that eyes-open resting-state alpha activity may be particularly sensitive to trauma-related alterations, providing a rationale for investigating alpha reactivity as a potential marker of state-dependent regulation following trauma.

Peak alpha frequency (PAF) characterises the temporal properties of alpha oscillations and has been proposed as an index of thalamocortical processing, attentional readiness, and aspects of arousal regulation (22, 23). PAF appears to represent a relatively stable neurophysiological characteristic in healthy adults and has been investigated across a range of neurological and psychiatric conditions (24). Higher PAF has been reported in individuals with PTSD and was modestly associated with both clinician- and self-rated PTSD symptom severity. Wahbeh and Oken (25) speculated that elevated PAF may reflect increased vigilance or cognitive preparedness, although these interpretations remain to be empirically established. Collectively, these findings suggest that PAF provides further insight into the functional characteristics of alpha oscillations, complementing measures of alpha amplitude and alpha reactivity. Whilst these indices describe distinct properties of alpha activity, EEG coherence extends this perspective by examining functional communication between cortical regions.

Functional connectivity metrics such as EEG coherence provide indirect measures of synchronisation and coupling between cortical regions, offering an index of large-scale neural communication and integration (26–28). Resting-state studies in PTSD have reported abnormalities in alpha-band connectivity and network organisation involving large-scale neural systems implicated in salience processing and self-regulation (18, 29, 30). Findings of both hyperconnectivity and dysconnectivity suggest that chronic trauma is characterised by disturbances in network organisation rather than uniform increases or decreases in cortical coupling. These observations suggest that EEG coherence offers a distinct perspective on large-scale neural communication alongside indices of alpha oscillatory activity. Given the central role of alpha oscillatory activity and functional connectivity in large-scale neural regulation, interventions capable of modifying these electrophysiological processes have attracted increasing interest.

Neurofeedback is a non-invasive psychophysiological intervention that provides individuals with real-time feedback of neural activity to facilitate self-regulation (31). Increasingly, neurofeedback is conceptualised not simply as a symptom-focused intervention but as an approach that aims to modify electrophysiological processes underlying self-regulation, neural plasticity, and large-scale brain network function, thereby supporting improvements across a broad range of trauma-related symptoms (32, 33). Consistent with this regulation-oriented framework, systematic reviews have demonstrated that neurofeedback is associated with improvements in PTSD symptoms, including reductions in hyperarousal and broader trauma-related symptomatology (32–34). Studies combining EEG neurofeedback with functional neuroimaging further demonstrate that modulation of alpha oscillations is accompanied by alterations in default mode and salience network connectivity, suggesting that alpha activity reflects large-scale neural regulation rather than local cortical activity alone (29, 30). For example, Nicholson et al. (30) demonstrated that alpha-rhythm neurofeedback altered amygdala connectivity patterns and increased post-training alpha synchronisation, accompanied by reductions in hyperarousal and PTSD symptom severity. These findings suggest that neurofeedback may facilitate adaptive modulation of large-scale neural systems supporting cortical inhibition, self-regulation, and arousal regulation.

Recent studies using portable EEG systems provide further evidence that alpha oscillatory activity can be modified through neurofeedback. In a small study of participants with chronic PTSD in Rwanda, du Bois et al. (35) reported increased resting-state alpha power following alpha-regulation training, accompanied by reductions in several measures of PTSD symptom severity. Portable alpha-neurofeedback studies in healthy young adults have similarly demonstrated modulation of alpha-band power following repeated training (36, 37). Although these studies differ substantially in population, neurofeedback protocol and study design, they provide converging evidence that alpha oscillatory dynamics are responsive to neurofeedback and support further investigation of resting-state alpha as a potential electrophysiological marker of treatment-associated change.

Previous work in traumatised refugee populations has demonstrated that neurofeedback is associated with improvements in trauma-related symptoms and clinical functioning, accompanied by normalisation of event-related potential (ERP) indices of attentional and cognitive processing (38, 39). Although these findings suggest that neurofeedback influences task-evoked neural processing, ERPs primarily reflect neural responses to specific cognitive events. By contrast, resting-state EEG characterises spontaneous neural oscillatory activity and intrinsic functional network organisation, providing insight into neural regulatory processes that may underlie treatment response. Examining changes in resting-state EEG may therefore contribute to understanding whether psychological recovery from chronic war-related trauma is accompanied by changes in cortical oscillatory activity and state-dependent regulation.

## 2 Study aims and hypotheses

### 2.1 Study aims

The present study investigated clinical and electrophysiological changes in adult refugees with persistent trauma-related psychopathology receiving multidisciplinary trauma treatment incorporating neurofeedback. Analyses were conducted within a transdiagnostic framework that focused on neural regulation across trauma-related disorders. The primary aims were to examine pre- to post-treatment changes in (a) overall clinical functioning and (b) resting-state alpha oscillatory activity and EEG coherence. Specifically, we examined changes in alpha amplitude, peak alpha frequency (PAF), alpha reactivity, and EEG coherence.

Secondary aims were to examine whether changes in electrophysiological measures were associated with overall clinical improvement and whether responders and non-responders differed in baseline electrophysiological characteristics and treatment-related electrophysiological change.

### 2.2 Hypotheses

#### 2.2.1 Primary hypothesis

Based on previous literature, we expected that neurofeedback within multidisciplinary care would be associated with clinically meaningful overall improvement, as measured by the Clinical Global Impression–Improvement (CGI-I) scale, together with increased resting-state alpha amplitude, increased alpha reactivity, and changes in peak alpha frequency and alpha-band coherence.

#### 2.2.2 Secondary hypotheses

Changes in resting-state electrophysiological measures would be associated with the degree of overall clinical improvement.

#### 2.2.3 Exploratory hypothesis

Responders and non-responders would differ in baseline electrophysiological characteristics and in patterns of electrophysiological change following neurofeedback.

## 3 Materials and methods

### 3.1 Study design

This retrospective observational pre-post study examined clinical and electrophysiological outcomes following neurofeedback in adult refugees receiving treatment at the Neurofeedback Clinic of the NSW Service for the Treatment and Rehabilitation of Torture and Trauma Survivors (STARTTS), Sydney, Australia. Clinical and resting-state EEG data routinely collected as part of standard clinical care were analysed to examine changes following neurofeedback.

The manuscript was retrospectively reviewed against the Consensus on the Reporting and Experimental Design of Clinical and Cognitive-Behavioural Neurofeedback Studies (CRED-nf) checklist (40). A completed CRED-nf checklist is provided in **Supplementary Table S6**. Reporting also followed the Strengthening the Reporting of Observational Studies in Epidemiology (STROBE) guidelines (41).

### 3.2 Participants

#### 3.2.1 Setting

STARTTS is a specialist multidisciplinary mental health service providing assessment and treatment for refugees and asylum seekers who have experienced torture, war, organised violence, persecution, and forced displacement. The Neurofeedback Clinic operates as a specialised service for clients who continue to experience significant trauma-related symptoms despite standard treatment.

This study included all consecutive eligible adult participants treated at the Neurofeedback Clinic between February 2017 and October 2020. Neurofeedback was offered as a second-line intervention to clients who continued to experience persistent trauma-related symptoms despite at least six months of previous psychological treatment and/or pharmacotherapy.

The cohort comprised adult refugees with chronic trauma-related psychopathology characterised by extensive psychiatric comorbidity and heterogeneous clinical presentations. Participants presented with varying combinations of posttraumatic stress, depression, anxiety, sleep disturbance, dissociative symptoms and somatic symptoms, reflecting the heterogeneity of trauma-related psychopathology encountered in routine clinical practice.

#### 3.2.2 Clinical assessment and diagnosis

All participants underwent comprehensive multidisciplinary assessment as part of routine clinical care at STARTTS. Assessments were conducted by registered psychologists and incorporated detailed clinical interviews, complemented by psychiatric and psychological reports, behavioural observations, and standardised self-report measures routinely administered within the service.

DSM-5 diagnoses were established through integration of multiple sources of clinical information, including structured clinical interviews, psychiatric assessments, standardised questionnaires, behavioural observations, and review of historical clinical documentation. Final diagnoses were confirmed through multidisciplinary case review involving psychologists and psychiatrists.

Although most participants met DSM-5 diagnostic criteria for PTSD, psychiatric comorbidity was common. Participants frequently met criteria for depressive disorders, anxiety disorders, sleep disorders, and other trauma-related presentations, reflecting the complexity of refugee mental health. Given the substantial overlap amongst these disorders in symptom presentation and disturbances in arousal regulation, emotional regulation, and cognitive functioning, the present study adopted a transdiagnostic framework focusing on overall clinical improvement and neural regulation rather than diagnosis-specific outcomes.

#### 3.2.3 Eligibility criteria

Participants were eligible if they were aged 21 years or older, had a refugee or asylum seeker background, had experienced war, torture, organised violence, persecution, or forced displacement, had completed both pre- and post-treatment resting-state EEG assessments, and had completed at least ten neurofeedback sessions.

Participants were excluded if they had previously received neurofeedback treatment, sustained a severe traumatic brain injury requiring hospitalisation, had neurological disorders likely to affect EEG interpretation, current substance-related disorders (excluding nicotine and caffeine), or required urgent medical intervention.

#### 3.2.4 Ethics

Participants provided written informed consent for the use of their clinical data for research during their initial EEG assessment. Ethical approval was obtained from the South Western Sydney Local Health District Human Research Ethics Committee (HREC No. LNR/15/LPOOL/369).

### 3.3 Neurofeedback intervention

Neurofeedback was delivered as part of routine multidisciplinary clinical care within the STARTTS Neurofeedback Clinic. Treatment consisted of one-hour sessions conducted once or twice weekly. Each session integrated approximately 10–20 minutes of neurofeedback with trauma counselling addressing both the physiological consequences of trauma exposure and ongoing post-migration stressors.

Neurofeedback was administered using EEGer-4 software (EEGer LLC, Granada Hills, CA, USA) with a J&J Spectrum four-channel amplifier (J404). Participants were seated in a comfortable recliner approximately 1.3 m from the feedback screen. Solid silver electrodes were applied at the relevant scalp and reference sites following skin preparation with NuPrep abrasive gel and using Ten20 conductive paste, with electrode impedances maintained below 5 kΩ. Visual and auditory feedback was generated in real time from participants’ EEG activity. The Formation game was used for most participants, with successful attainment of reinforcement criteria progressively uncovering a selected image on the screen. Visual material included nature scenes, animals and, where appropriate, photographs chosen or supplied by participants. Images could be changed according to participant preference, including where particular content elicited discomfort or distress. Achievement of training criteria was also accompanied by auditory tones, providing concurrent auditory reinforcement. Reinforcement thresholds were generally maintained between 60% and 70% and adjusted dynamically throughout training to optimise learning whilst maintaining participant comfort.

Treatment protocols, including electrode placement and reinforcement frequencies, were individualised according to each participant’s presenting symptoms, predominant pattern of over- or under-arousal, arousal stability and response to training. Interhemispheric protocols were initially selected for participants with marked instability (i.e. panic attacks), whilst right hemispheric protocols were used to address persistent hyperarousal symptoms such as anxiety, fear and insomnia. Posterior protocols were incorporated in participants presenting with heightened sensory and environmental reactivity, whilst central and fronto-parietal protocols were used where attentional and regulatory difficulties persisted. Electrode placements included interhemispheric temporal and central montages (T3–T4, T5–T6 and C3–C4); predominantly right-sided temporal, central and parietal montages (T4–P4, C4–A2, C4–P4, C4–Pz and Cz–T6); posterior midline montages (Pz–A2 and Iz–A2); a central midline montage (Cz–A2); a fronto-parietal midline montage (Fz–Pz); and an individualised left centro-temporal montage (Cz–T5).

Electrode placements and reinforcement frequencies were reviewed continuously throughout treatment and modified according to changes in symptoms, participant feedback, therapist observations, functional improvement, and overall regulatory stability, reflecting the adaptive regulation-oriented neurofeedback model routinely employed within the STARTTS Neurofeedback Clinic. Protocol modifications reflected ongoing clinical reassessment rather than symptom matching alone.

Neurofeedback and trauma counselling were delivered by the same clinician to facilitate integration of physiological regulation with psychological treatment. All clinicians were registered psychologists certified in neurofeedback through the Biofeedback Certification International Alliance (BCIA) and received regular supervision from senior neurofeedback clinicians. Trauma counselling followed culturally informed, trauma-focused approaches tailored to each participant’s clinical presentation and refugee experience. Accredited interpreters were utilised whenever required.

The number of treatment sessions was determined according to clinical need, treatment response and participant preference, consistent with routine clinical practice.

Neurofeedback protocol characteristics were summarised across all available treatment sessions. For each protocol application, electrode placement, reward frequency range and the corresponding number of protocol applications were extracted. Mean reward frequencies were calculated as the midpoint of the documented minimum and maximum reward frequencies and weighted by the number of protocol applications. Placement frequencies were summarised as the number and proportion of participants receiving each placement and the number and proportion of protocol applications across the entire treatment period. The proportion of protocol applications targeting the alpha range was calculated from the documented reward frequency ranges. Further details of the neurofeedback protocol analysis are provided in **Supplementary Methods S1**.

### 3.4 Clinical outcome measures

The primary clinical outcome was overall clinical improvement assessed using the Clinical Global Impression (CGI) scale. The CGI was selected because it provides a brief, validated clinician-rated measure of illness severity and treatment response that is applicable across psychiatric diagnoses and is well suited to clinical settings (42).

All participant records were independently rated by two registered psychologists using the CGI-I, based on documented changes in symptoms, functioning and overall clinical presentation across the treatment period. Although raters had access to information regarding clinical progress contained in the treatment records, they had no knowledge of the pre-post EEG changes analysed in the present study or of the study hypotheses. Where the two raters did not agree, the case was reviewed with a third assessor, a psychiatrist, to reach consensus. The original independent ratings were not retained separately from the final consensus ratings; consequently, inter-rater reliability could not be calculated retrospectively.

Two CGI domains were used, following the operational definitions described by Busner and Targum (43). Severity of Illness (CGI-S) was used to rate overall clinical severity at baseline and following completion of treatment on a seven-point scale ranging from 1 (normal, not at all ill) to 7 (amongst the most extremely ill patients). Clinical Global Improvement (CGI-I) was used to evaluate overall change following neurofeedback, with scores ranging from 1 (very much improved) to 7 (very much worse); a score of 4 indicated no change. Detailed rating criteria are provided in **Supplementary Table S1**.

### 3.5 EEG recording

Resting-state EEG recordings were acquired before the commencement of neurofeedback and following completion of treatment using a Mitsar-201 amplifier (Mitsar Co., St Petersburg, Russia). Nineteen Ag/AgCl electrodes were positioned according to the International 10–20 System (Fp1, Fp2, F7, F3, Fz, F4, F8, T3, C3, Cz, C4, T4, T5, P3, Pz, P4, T6, O1, and O2). Recordings were obtained in a quiet testing room with participants seated in a comfortable recliner approximately 1m from the screen and instructed to minimise movement. Electrodes were applied following skin preparation with NuPrep abrasive gel, with Electro-gel used to ensure adequate electrical conductivity. EEG was recorded during 10-minute eyes-open and 10-minute eyes-closed resting-state conditions under standardised recording procedures. Signals were sampled at 500 Hz, and electrode impedances were maintained below 5 kΩ throughout the recording. EEG assessments were generally conducted before 2:00 pm to minimise potential effects of fatigue; however, the recording times were not systematically matched between pre- and post-treatment assessments. Caffeine and nicotine consumption prior to EEG recording were routinely recorded as part of the clinical assessment but were not systematically restricted or examined in the present retrospective analysis.

### 3.6 EEG processing

EEG data were processed offline using a custom analysis pipeline implemented in MATLAB (R2025b; MathWorks, Natick, MA) with the EEGLAB toolbox (version 2026.0.0; 44). Raw EDF files were imported using the BioSig toolbox (45). Non-EEG auxiliary channels were removed.

Continuous EEG data were band-pass filtered between 0.5 and 45 Hz using a zero-phase finite impulse response filter. Line noise was attenuated using a 50 Hz notch filter with a 2 Hz bandwidth. For independent component analysis (ICA) decomposition, data were additionally high-pass filtered at 1.0 Hz and low-pass filtered at 40 Hz to optimise component estimation.

ICA decomposition was performed using cuDaICA, a GPU-accelerated implementation of the Infomax algorithm (46). Data were resampled to 250 Hz prior to ICA to reduce computational demands. Artifactual components were identified and removed using ICLabel (47), with automatic rejection thresholds set at 0.8 probability for eye, muscle, heart, line noise, and channel noise components. ICLabel classification required a minimum of four channels with valid three-dimensional coordinates.

Bad channels were identified using a correlation-based criterion (threshold = 0.65) and flatline detection (5-second threshold). Channels identified as bad were interpolated using spherical spline interpolation, provided that fewer than 20% of channels (approximately 3–4 of 19 channels) were flagged; otherwise, bad channel removal was skipped to preserve data integrity. Following artefact correction, data were re-referenced to the average of all scalp electrodes. Only artefact-free EEG segments were retained for quantitative analyses.

### 3.7 EEG outcome measures

Primary electrophysiological outcomes were selected *a priori* to characterise different aspects of resting-state alpha oscillatory activity and large-scale neural communication. The alpha band was defined as 8–13 Hz, consistent with standard definitions in the EEG literature (15).

#### 3.7.1 Peak alpha frequency

Global peak alpha frequency (PAF) was calculated as the centre-of-gravity frequency within the alpha band (8–13 Hz) across all 19 scalp channels. Power spectral density (PSD) was estimated using the multi-taper method with discrete prolate spheroidal sequences (DPSS; 48). A time-bandwidth product of 3 and 5 tapers was used, with a maximum FFT length of 216 points (65,536 samples), yielding a frequency resolution of approximately 0.008 Hz at a sampling rate of 500 Hz. PAF was computed as the power-weighted mean frequency:


PAF=∑(f×PSD(f))/∑PSD(f)

where f denotes frequency bins within the alpha range and PSD(f) denotes the corresponding power spectral density values (49).

#### 3.7.2 Alpha amplitude

Global alpha amplitude was quantified as alpha-band power (μV^2^) across all 19 scalp electrodes within the 8–13 Hz alpha band. Alpha-band power was calculated by integrating the power spectral density (PSD) across the alpha frequency range using trapezoidal numerical integration. For consistency with the terminology used throughout this manuscript, this measure is referred to as alpha amplitude. Global alpha amplitude was defined as the arithmetic mean of alpha-band power across all scalp electrodes. For analyses of alpha reactivity, alpha band power was also calculated separately for the eyes-open and eyes-closed resting-state conditions.

#### 3.7.3 Alpha reactivity

Alpha reactivity was calculated as the natural logarithm of the ratio of alpha power during eyes-closed versus eyes-open conditions:


$$Alpha\;Reactivity=\ln\left(\alpha_{EC}\right)-\ln\left(\alpha_{EO}\right)$$

This log-ratio metric provides a normalised measure of state-dependent modulation of alpha oscillatory activity that is robust to individual differences in absolute alpha power (20). Positive values indicate greater alpha power during eyes-closed than eyes-open resting conditions, reflecting the normal attenuation (“alpha blocking”) of alpha activity following eye opening. Lower values therefore indicate a smaller difference between eyes-closed and eyes-open alpha activity.

Occipital alpha reactivity was calculated using a region of interest (ROI) comprising electrodes O1, O2, and Pz, consistent with the posterior distribution of the dominant alpha rhythm during resting wakefulness (15). Frontal alpha reactivity was calculated using an ROI comprising electrodes F3, F4, and Fz.

Alpha reactivity was treated as the primary measure of state-dependent alpha modulation. Because occipital alpha reactivity showed a clear departure from normality, pre- to post-treatment change was evaluated using the Wilcoxon signed-rank test. The previously reported parametric Time × Condition analysis of the same underlying contrast was removed to avoid applying different inferential tests to closely related contrasts.

#### 3.7.4 EEG coherence

Functional connectivity was assessed using magnitude-squared coherence, a frequency-domain measure of linear coupling between electrode pairs (50). Coherence was computed using Welch’s averaged periodogram method with a 2-second Hann window and 50% overlap.

Interhemispheric coherence was calculated as the mean alpha-band coherence across three homologous electrode pairs: C3–C4 (central), P3–P4 (parietal), and O1–O2 (occipital). This composite metric provides an index of interhemispheric communication across central, parietal, and occipital regions.

Anterior–posterior coherence was calculated as alpha-band coherence between Fz (midline frontal) and Pz (midline parietal) electrodes, providing an index of long-range cortical connectivity along the anterior–posterior axis.

Raw coherence values (ranging from 0 to 1) were used in all analyses without transformation, as coherence values are bounded and do not require normalisation for parametric statistical testing when used as dependent variables (50).

The selected electrophysiological outcomes were chosen to characterise complementary aspects of neural regulation. Global alpha amplitude, peak alpha frequency, and alpha reactivity indexed different properties of resting-state alpha oscillatory activity, whereas EEG coherence provided measures of large-scale functional connectivity. These complementary measures enabled evaluation of both local oscillatory dynamics and distributed neural communication following neurofeedback.

### 3.8 Statistical analysis

Statistical analyses were performed using Python (version 3.11) with SciPy (version 1.11; 51), statsmodels (version 0.14; 52), NumPy (53), and pandas (54). Figures were generated using Matplotlib (55).

As this was a retrospective observational study of routinely collected clinical data, no *a priori* sample size calculation was performed. The sample comprised all consecutive eligible participants with complete pre- and post-treatment EEG and clinical outcome data during the study period.

For the available sample of N = 53, a paired comparison at α = .05 (two-tailed) had 80% power to detect an effect of approximately d = 0.39. The study therefore had limited sensitivity to smaller effects, particularly for secondary outcomes and subgroup analyses. Accordingly, null findings for secondary electrophysiological outcomes and subgroup analyses should be interpreted cautiously, and greater emphasis should be placed on the magnitude and precision of observed effects rather than statistical significance alone.

To support an estimation-focused interpretation, effect-size estimates are reported alongside the corresponding statistical tests. Cohen’s d is reported for outcomes analysed parametrically, whilst rank-biserial correlation is reported for occipital alpha reactivity, which was analysed using the Wilcoxon signed-rank test because of marked skewness and influential outlying values. Confidence intervals are reported for the principal effects where applicable.

Descriptive statistics summarised demographic, clinical, treatment, and electrophysiological characteristics. The primary clinical outcome was overall clinical improvement measured using the CGI. Participants were classified as responders (CGI-I ≤ 2) or non-responders (CGI-I ≥ 3) based on established clinical conventions.

#### 3.8.1 Primary analyses

Changes in EEG measures were assessed using a distribution-informed approach. Distributional assumptions were evaluated using Shapiro–Wilk tests together with inspection of the shape of the change-score distributions, including skewness and potentially influential observations. Preliminary normality testing alone was not used as a mechanical rule for choosing between parametric and non-parametric tests, because such two-stage procedures can themselves affect statistical inference and t-tests are reasonably robust to some departures from normality (56, 57). Accordingly, paired t-tests were retained for global alpha amplitude, global peak alpha frequency, interhemispheric coherence, and anterior–posterior coherence, with Cohen’s d reported as the standardised estimate of mean change.

Occipital alpha reactivity was treated differently because its change-score distribution showed marked positive skewness (skewness = 1.99) and influential outlying values, and sensitivity analyses produced materially different parametric and rank-based inferences (paired t-test p = .097; Wilcoxon p = .006). In this setting, the mean change was not considered an adequate summary of the typical within-participant shift. The Wilcoxon signed-rank test was therefore designated the primary analysis for occipital alpha reactivity, with rank-biserial correlation and its 95% confidence interval reported as the corresponding effect-size estimate. This outcome-specific decision was based on the observed distributional characteristics and robustness of inference rather than on the result of the Shapiro–Wilk test alone.

To account for multiple comparisons, p-values were adjusted using the Benjamini–Hochberg false discovery rate (FDR) procedure (58). Primary outcomes (global PAF, global alpha amplitude, interhemispheric coherence, and anterior–posterior coherence) were corrected together as a family of four tests. Secondary outcomes (occipital and frontal alpha reactivity) were corrected separately as a family of two tests to maintain statistical power whilst controlling the expected proportion of false discoveries.

#### 3.8.2 Alpha reactivity analysis

The primary alpha-reactivity analysis compared pre- and post-treatment occipital alpha-reactivity values using the Wilcoxon signed-rank test. The redundant parametric Time × Condition ANOVA previously used to characterise the same contrast was removed. Descriptive eyes-open and eyes-closed alpha values are retained to illustrate the direction of change across recording conditions. For exploratory response-group analyses, the responder versus non-responder interaction contrast was compared directly using an independent-samples t-test; a Mann–Whitney sensitivity analysis yielded the same non-significant conclusion.

Within-condition eyes-open and eyes-closed values are presented descriptively to characterise the pattern underlying alpha reactivity; inferential interpretation is based on the primary alpha-reactivity test and, for response-group comparisons, the direct between-group interaction contrast.

#### 3.8.3 Response group comparisons

Baseline electrophysiological differences between responders and non-responders were assessed using independent-samples Wilcoxon rank-sum tests (Mann–Whitney U tests). Pre- to post-treatment changes within each response group were assessed using Wilcoxon signed-rank tests with FDR correction applied separately within each group.

#### 3.8.4 Correlation analyses

Associations between changes in EEG measures and overall clinical improvement (CGI change score) were examined using Spearman’s rank correlation coefficients (ρ). Spearman correlations were selected given the ordinal nature of the CGI change score and potential non-linearity in brain–behaviour relationships. Correlation p-values were adjusted using the Benjamini–Hochberg FDR procedure, with primary and secondary metrics corrected as separate families.

#### 3.8.5 Missing data

Participants were included in analyses if they had complete pre- and post-treatment EEG data. The final analytical sample comprised 53 participants with complete EEG and CGI-I data. Of these, 38 (72%) were assessed at DONE 2 and 15 (28%) at DONE 3. The mean interval between baseline and final EEG assessment was 435 days (SD = 272; range = 87–1367 days). For participants whose final assessment was DONE 2, the mean interval was 375 days (SD = 210; range = 87–998), compared with 586 days (SD = 353; range = 134–1367) for those assessed at DONE 3. Reasons for missing follow-up EEG were not systematically recorded and therefore could not be reliably determined retrospectively. Post-treatment EEG assessments preferentially used DONE 3 where available; otherwise, DONE 2 was used. Sensitivity analyses restricted to DONE 2 were conducted to assess whether variation in assessment endpoint materially influenced the principal findings.

#### 3.8.6 Significance thresholds

Statistical significance was set at p <.05 (two-tailed) following FDR correction. Effect sizes were interpreted according to conventional benchmarks: small (d = 0.20), medium (d = 0.50), and large (d = 0.80; 59).

## 4 Results

### 4.1 Participant flow and characteristics

Participant flow through the study is presented in **Figure 1**. Between February 2017 and October 2020, 81 potentially eligible patients were identified. Of these, 77 had baseline EEG recordings, 60 had follow-up EEG recordings, 56 had matched pre–post recordings, and 53 met the final inclusion criteria and had complete clinical outcome data. Reasons for missing follow-up EEG were not systematically recorded and therefore cannot be reliably determined retrospectively. All 53 participants were included in responder and non-responder subgroup analyses.

**Figure 1:**
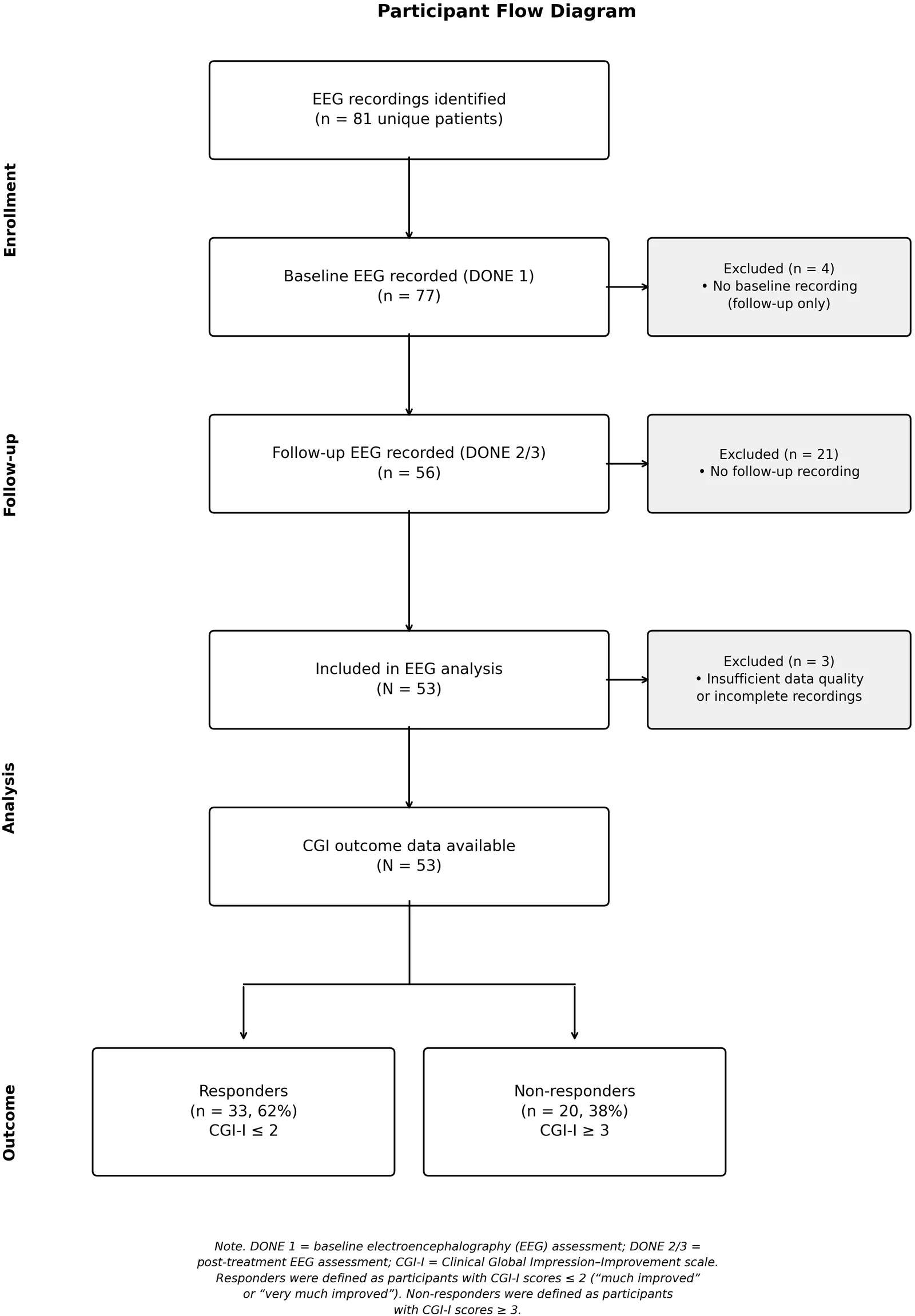
Participant flow diagram. DONE 1, baseline electroencephalography (EEG) assessment; DONE 2/3, post-treatment EEG assessment; CGI-I, Clinical Global Impression–Improvement scale. Responders were defined as participants with CGI-I scores ≤ 2 (“much improved” or “very much improved”). Non-responders were defined as participants with CGI-I scores ≥ 3.

Demographic and clinical characteristics are summarised in **Table 1**. Participants had a mean age of 47.9 years (SD = 9.7; range: 23–65), and 25 (47%) were female. The cohort predominantly comprised refugees from the Middle East, with most participants born in Iraq (68%) or Iran (11%). The majority were permanent residents or Australian citizens (85%), and 74% required interpreter support during assessment and treatment.

**Table 1:** Demographic, clinical, and treatment characteristics of the study sample.

Characteristic	Overall Sample (N = 53)
Demographic characteristics	
Age, years, mean ± SD (range)	47.9 ± 9.7 (23–65)
Female, n (%)	25 (47)
Residential status, n (%)	
Australian citizen	7 (13)
Permanent resident	38 (72)
Temporary visa	2 (4)
Asylum seeker	6 (11)
Country of birth, n (%)	
Iraq	36 (68)
Iran	6 (11)
Other†	11 (21)
Interpreter required, n (%)	39 (74)
Marital status, n (%)	
Married	32 (60)
Never married	8 (15)
Divorced	5 (9)
Widowed	5 (9)
Separated	1 (2)
Not stated	2 (4)
Household size, mean ± SD	3.0 ± 1.6
Educational level, n (%)	
Tertiary (Diploma or higher)	11 (21)
Vocational certificate	2 (4)
Secondary school	20 (38)
Primary school	11 (21)
No formal schooling	2 (4)
Not stated	7 (13)
Clinical characteristics	
Baseline CGI-Severity, mean ± SD	4.6 ± 0.6
Number of comorbid diagnoses, mean ± SD	4.7 ± 1.2
Trauma exposure, n (%)	
Torture	28 (53)
Witnessing atrocities	27 (51)
Childhood trauma	20 (38)
Psychotropic medication, n (%)	41 (77)
Medication classes, n (%)	
SSRI	14 (26)
Anticonvulsant / mood stabiliser	12 (23)
Atypical antidepressant	11 (21)
SNRI	10 (19)
Benzodiazepine / anxiolytic	8 (15)
Treatment characteristics	
Trauma counselling sessions before neurofeedback, mean ± SD	36.7 ± 26.9
Neurofeedback sessions, mean ± SD (range)	38.7 ± 21.3 (17–114)
Treatment duration, months, mean ± SD	10.2 ± 8.4

Values are presented as mean ± SD unless otherwise indicated. Categorical variables are presented as n (%). Medication classes are not mutually exclusive; participants could receive more than one medication.

CGI, Clinical Global Impression; SD, standard deviation; SSRI, selective serotonin reuptake inhibitor; SNRI, serotonin–noradrenaline reuptake inhibitor.

† Other countries: Afghanistan, Bosnia and Herzegovina, Eritrea, Ethiopia, Lebanon, Liberia, Sierra Leone, Somalia, Sudan, Syria, and Turkey.

The cohort was characterised by chronic trauma-related psychopathology with substantial psychiatric complexity. Baseline illness severity was moderate to severe, with a mean CGI-Severity score of 4.5 (SD = 0.6), and 51% of participants were rated as markedly ill or more severely ill. Participants reported extensive trauma exposure, including torture (53%), witnessing atrocities (51%), and childhood trauma (38%), and had a mean of 4.7 (SD = 1.2) DSM psychiatric diagnoses. Trauma- and stressor-related disorders were present in all participants. The most common comorbid diagnostic categories were depressive disorders (94%), sleep-wake disorders (89%), somatic symptom disorders (66%), anxiety disorders (57%), and dissociative disorders (25%), with smaller proportions meeting criteria for persistent complex bereavement disorder (19%), substance-related disorders (15%), personality disorders (11%), and bipolar disorder (2%). Most participants (77%) were prescribed psychotropic medication at the time of assessment.

Before commencing neurofeedback, the participants had received a mean of 36.7 trauma counselling sessions (SD = 26.9) at STARTTS, indicating persistent trauma-related symptoms despite substantial previous psychological treatment.

### 4.2 Neurofeedback intervention characteristics

Participants completed a mean of 38.7 neurofeedback sessions (SD = 21.3; range = 17–114) over a mean treatment duration of 10.2 months (SD = 8.4). Neurofeedback was delivered using an individualised, regulation-oriented treatment model in which protocols were modified throughout therapy according to participants’ clinical presentation, treatment response, participant feedback, and therapist observations.

Participants received a mean of 2.58 different neurofeedback protocols (SD = 0.93), with 92.5% receiving more than one protocol during treatment. Interhemispheric protocols were the most frequently utilised (88.7% of participants), followed by right hemisphere (81.1%) and central protocols (47.2%). Posterior (17.0%), fronto-parietal (15.1%), and individualised left temporal protocols (9.4%) were used less frequently.

Across all recorded protocol applications, interhemispheric protocols accounted for 41.8% of protocol use, followed by right hemisphere (36.1%) and central protocols (11.9%). Posterior, fronto-parietal, and individualised left temporal protocols together accounted for the remaining 10.2% of protocol applications. The most frequently utilised electrode placements were T3–T4, T4–P4, Cz–A2, and C3–C4 (**Table 2**; **Supplementary Table S2**).

**Table 2:** Characteristics of neurofeedback protocol implementation across 2,361 protocol applications.

Electrode placement	Participants, n (%)	Protocol applications, n (%)	Session-weighted mean reward frequency (Hz)
T3–T4	42 (79.2)	722 (30.6)	11.71
T4–P4	34 (64.2)	513 (21.7)	10.09
T5–T6	12 (22.6)	139 (5.9)	11.19
C3–C4	25 (47.2)	214 (9.1)	12.54
C4–A2	12 (22.6)	49 (2.1)	11.96
C4–P4	7 (13.2)	19 (0.8)	11.15
C4–Pz	7 (13.2)	68 (2.9)	11.79
Cz–A2	25 (47.2)	251 (10.6)	13.27
Cz–T5	5 (9.4)	33 (1.4)	13.05
Cz–T6	13 (24.5)	174 (7.4)	13.23
Pz–A2	7 (13.2)	111 (4.7)	9.82
Iz–A2	2 (3.8)	13 (0.6)	7.08
Fz–Pz	8 (15.1)	55 (2.3)	13.35
Overall	53	2,361 (100)	11.63

Participants could receive more than one electrode placement during treatment; therefore, participant percentages do not sum to 100%.

Protocol characteristics were examined across all available treatment sessions from the 53 participants. Despite substantial variation in electrode placements and protocol progression, reinforcement frequencies showed relatively little variation throughout treatment. The overall session-weighted mean reward frequency was 11.63 Hz. Although mean reward frequencies for individual electrode placements ranged from 7.08 Hz (Iz–A2) to 13.35 Hz (Fz–Pz), 95.8% of protocol-specific reward-frequency ranges overlapped the 10–13 Hz alpha band. These findings suggest that treatment individualisation occurred primarily through the selection and progression of electrode placements, whereas reinforcement frequencies remained relatively stable and were predominantly centred within the alpha range. Amongst treatment records in which session duration was documented, active neurofeedback training typically lasted approximately 13–16 minutes per electrode placement. Additional details of the neurofeedback protocol analysis are provided in the **Supplementary Methods S1**. Detailed summaries of reward-frequency distributions, electrode placement analyses, and training duration are presented in **Supplementary Tables S3–S5** and **Supplementary Figures S1, S2**.

### 4.3 Clinical outcomes

Overall clinical improvement following neurofeedback was assessed using the Clinical Global Impression–Improvement (CGI-I) scale. Based on CGI-I ratings, 33 participants (62%) were classified as responders, demonstrating much improved or very much improved clinical status (CGI-I ≤ 2), whereas 20 participants (38%) were classified as non-responders (CGI-I ≥ 3).

Baseline demographic, clinical, and treatment characteristics of responders and non-responders are summarised in **Table 3**. The two groups did not differ significantly with respect to age, sex, marital status, household size, country of birth, baseline illness severity, number of comorbid diagnoses, torture exposure, witnessing atrocities, psychotropic medication use, treatment duration, prior counselling sessions, or the number of neurofeedback sessions completed (all p >.05). However, responders had significantly higher education levels (Mann–Whitney *U* = 195, *p* = .011), were less likely to require interpreter services (Fisher’s exact *p* = .009), and more frequently reported early childhood trauma (Fisher’s exact *p* = .009) than non-responders.

**Table 3:** Demographic, clinical, and treatment characteristics by clinical response status.

Variable	Responders (n = 33)	Non-responders (n = 20)	U or χ^2^	p
Demographics				
Age (years), M ± SD	47.4 ± 10.3	48.8 ± 8.8	302	.620
Female, n (%)	18 (55)	7 (35)	1.91	.167
Married, n (%)	18 (55)	14 (70)	1.24	.265
Household size, M ± SD	2.9 ± 1.5	3.1 ± 1.7	304	.633
Education level, Mdn (IQR)	Secondary (Bachelor–Secondary)	Primary (Secondary–Primary)	195	**.011**
Country of birth: Iraq, n (%)	22 (67)	14 (70)	0.06	.801
Interpreter required, n (%)	20 (61)	19 (95)	Fisher	**.009**
Clinical characteristics				
CGI-Severity baseline, M ± SD	4.5 ± 0.6	4.5 ± 0.7	340	.853
Number of comorbid diagnoses, M ± SD	4.8 ± 1.1	4.7 ± 1.4	348	.747
Torture exposure, n (%)	17 (52)	11 (55)	0.06	.805
Witnessed atrocities, n (%)	17 (52)	10 (50)	0.01	.915
Early childhood trauma, n (%)	17 (52)	3 (15)	Fisher	**.009**
Psychotropic medication, n (%)	26 (79)	15 (75)	0.10	.749
Treatment characteristics				
Neurofeedback sessions, M ± SD	40.3 ± 20.7	36.0 ± 22.5	358	.236
Treatment duration (months), M ± SD	9.9 ± 8.1	10.7 ± 9.1	292	.929
Prior counselling sessions, M ± SD	35.2 ± 22.1	39.2 ± 34.3	283	.790

Responders were defined as participants with CGI-Improvement ratings ≤ 2 (much improved or very much improved). Continuous variables are presented as M ± SD and compared using Mann–Whitney U tests. Categorical variables are presented as n (%) and compared using chi-square tests. Education level is presented as median (interquartile range) due to ordinal scaling. CGI, Clinical Global Impression. Bold p-values indicate statistical significance (p <.05).

To investigate whether electrophysiological characteristics differed according to clinical outcome, subsequent analyses compared responders and non-responders with respect to baseline resting-state EEG measures and treatment-related changes in alpha oscillatory activity and functional connectivity.

### 4.4 Changes in resting-state alpha oscillatory activity

Pre- to post-treatment changes in resting-state alpha oscillatory activity are illustrated in **Figure 2**. The analyses examining eyes-open and eyes-closed alpha dynamics underlying alpha reactivity are presented in **Figure 3**.

**Figure 2:**
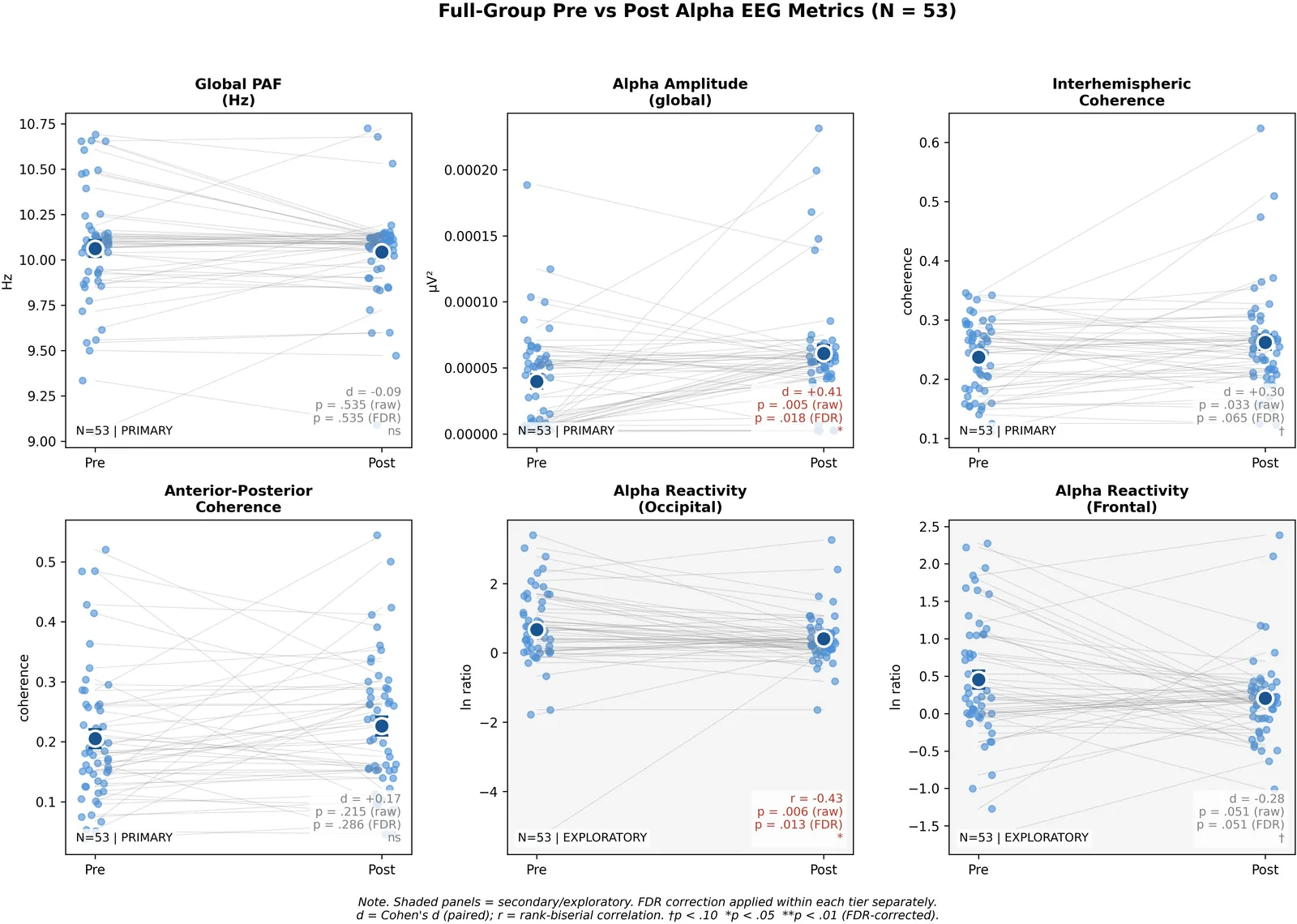
Full-group pre- versus post-treatment alpha EEG metrics (*N* = 53). Each panel shows individual participant trajectories (thin grey lines) connecting pre- and post-treatment values, with group means (large circles) and standard errors. Effect sizes, raw p-values, and FDR-corrected p-values are displayed for each metric. Cohen’s d represents the paired-samples effect size; r represents the rank-biserial correlation. Shaded panels indicate exploratory outcomes. FDR correction was applied separately to primary and exploratory outcomes. †p <.10, *p <.05 (FDR-corrected).

**Figure 3:**
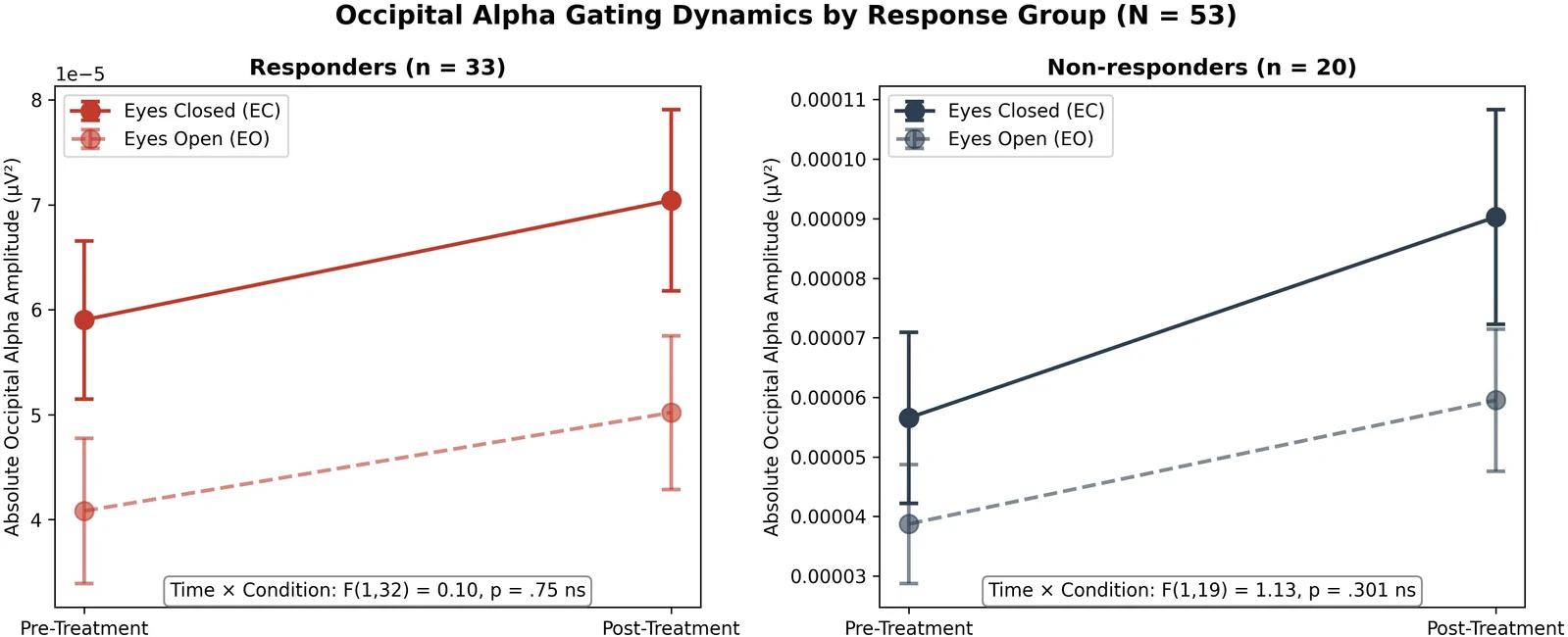
Mean absolute occipital alpha amplitude (μV^2^) during eyes-closed (EC; solid line) and eyes-open (EO; dashed line) conditions at pre- and post-treatment timepoints for responders (n = 33; left panel) and non-responders (n = 20; right panel). Error bars represent standard errors of the mean. The direct responder versus non-responder comparison of the interaction contrast was t(51) = 0.88, p = .381, d = 0.24; Mann–Whitney sensitivity analysis, U = 350, p = .720.

#### 4.4.1 Global alpha amplitude

Neurofeedback was associated with a significant increase in global alpha amplitude across the full cohort (N = 53). Mean alpha amplitude increased from 3.98 × 10^−5^ μV^2^ (SD = 3.78 × 10^−5^) at baseline to 6.11 × 10^−5^ μV^2^ (SD = 4.36 × 10^−5^) following treatment, representing a small-to-medium effect size (d = 0.41, 95% CI [0.13, 0.69]). This increase remained statistically significant following false discovery rate (FDR) correction (pFDR = .018).

#### 4.4.2 Peak alpha frequency

Peak alpha frequency (PAF) remained stable following neurofeedback. Mean PAF changed minimally from 10.06 Hz (SD = 0.33) at baseline to 10.04 Hz (SD = 0.25) at post-treatment, with a negligible effect size (d = −0.09) that was not statistically significant following FDR correction (pFDR =.535).

#### 4.4.3 Alpha reactivity

Occipital alpha reactivity decreased from 0.68 (SD = 1.33) at baseline to 0.41 (SD = 0.72) at post-treatment. Because the change-score distribution was markedly positively skewed and contained influential outlying values, the Wilcoxon signed-rank test was used as the primary inferential analysis. This indicated a significant reduction in occipital alpha reactivity (W = 408, p = .006; FDR-adjusted p = .013; rank-biserial r = −0.43, 95% CI [−0.69, −0.13]), with 66% of participants showing reduced reactivity. The corresponding descriptive Cohen’s d was −0.23.

Descriptive examination of occipital alpha amplitude showed increases in both recording conditions. Eyes-open alpha amplitude increased from 4.00 × 10^−5^ (SD = 4.12 × 10^−5^) to 5.37 × 10^−5^ μV^2^ (SD = 4.63 × 10^−5^; *d* = 0.25), whilst eyes-closed alpha amplitude increased from 5.81 × 10^−5^ (SD = 5.16 × 10^−5^) to 7.79 × 10^−5^ μV^2^ (SD = 6.31 × 10^−5^; *d* = 0.29). These condition-specific values are presented descriptively to characterise the changes underlying alpha reactivity; inferential interpretation of state-dependent modulation is based on the primary alpha-reactivity analysis.

Frontal alpha reactivity showed a similar descriptive reduction, decreasing from 0.45 (SD = 0.85) to 0.21 (SD = 0.57; *d* = −0.28), but this change did not reach statistical significance (raw and FDR-adjusted *p* = .051).

### 4.5 Changes in functional connectivity

Pre- to post-treatment changes in alpha-band functional connectivity are summarised in **Table 4** and illustrated in **Figure 2**.

**Table 4:** Pre- to post-treatment changes in resting-state alpha EEG measures.

Outcome	Pre-treatment M (SD)	Post-treatment M (SD)	Effect size	p	pFDR
Primary outcomes					
Global alpha amplitude (μV^2^)	3.98 × 10^−5^ (3.78 × 10^−5^)	6.11 × 10^−5^ (4.36 × 10^−5^)	d = 0.41	.005	**.018**
Global PAF (Hz)	10.06 (0.33)	10.04 (0.25)	d = −0.09	.535	.535
Interhemispheric coherence	0.237 (0.062)	0.263 (0.088)	d = 0.30	.033	.065
Anterior–posterior coherence	0.205 (0.113)	0.226 (0.112)	d = 0.17	.215	.286
Exploratory outcomes					
Occipital alpha reactivity	0.68 (1.33)	0.41 (0.72)	r = −0.43	.006	**.013**
Frontal alpha reactivity	0.45 (0.85)	0.21 (0.57)	d = −0.28	.051	.051

Values are presented as mean (SD). Paired *t*-tests were used for global alpha amplitude, global peak alpha frequency (PAF), interhemispheric coherence, anterior–posterior coherence, and frontal alpha reactivity, with Cohen’s *d* reported as the corresponding effect-size estimate. Occipital alpha reactivity was analysed using the Wilcoxon signed-rank test, with rank-biserial correlation (*r*) reported as the corresponding effect-size estimate. Raw *p*-values were adjusted using the Benjamini–Hochberg false discovery rate (FDR) procedure within prespecified tiers, with the four primary outcomes corrected together and the two exploratory outcomes corrected separately. Bold *p*FDR values indicate statistical significance following FDR correction (*p* <.05).

Both coherence measures demonstrated modest increases following neurofeedback; however, neither reached statistical significance following FDR correction.

#### 4.5.1 Interhemispheric coherence

Interhemispheric alpha coherence increased from 0.237 (SD = 0.062) at baseline to 0.263 (SD = 0.088) following treatment, representing a small-to-medium effect size (d = 0.30). The unadjusted paired t-test was nominally significant (p = .033), but this increase did not remain statistically significant following FDR correction (pFDR = .065).

#### 4.5.2 Anterior–posterior coherence

Anterior–posterior alpha coherence increased modestly from 0.205 (SD = 0.113) at baseline to 0.226 (SD = 0.112) following treatment, corresponding to a small effect size (d = 0.17). This change was not statistically significant (raw p = .215; FDR-adjusted p = .286).

Sensitivity analyses demonstrated that the primary and secondary EEG findings were robust to the exclusion of potential outliers, with no changes to the overall interpretation of the results (**Supplementary Material S1**).

A further sensitivity analysis examined whether variation in post-treatment assessment endpoint influenced the principal findings. Amongst participants assessed at DONE 2 (n = 38), global alpha amplitude increased (d = 0.38, p = .009), occipital alpha reactivity decreased (d = −0.25, p = .007), and global PAF remained unchanged (d = −0.06, p = .570). This pattern was consistent with the full-sample analysis, indicating that the principal findings were not materially altered by inclusion of participants assessed at DONE 3.

### 4.6 Electrophysiological characteristics of responders and non-responders

#### 4.6.1 Baseline electrophysiological characteristics

Baseline electrophysiological measures were compared between responders (n = 33) and non-responders (n = 20) to examine whether resting-state EEG characteristics differed according to subsequent clinical outcome (**Figure 4**). Descriptively, responders exhibited higher baseline occipital alpha reactivity than non-responders (0.78 vs 0.50), whereas non-responders exhibited higher baseline anterior–posterior alpha coherence than responders (0.238 vs 0.186).

**Figure 4:**
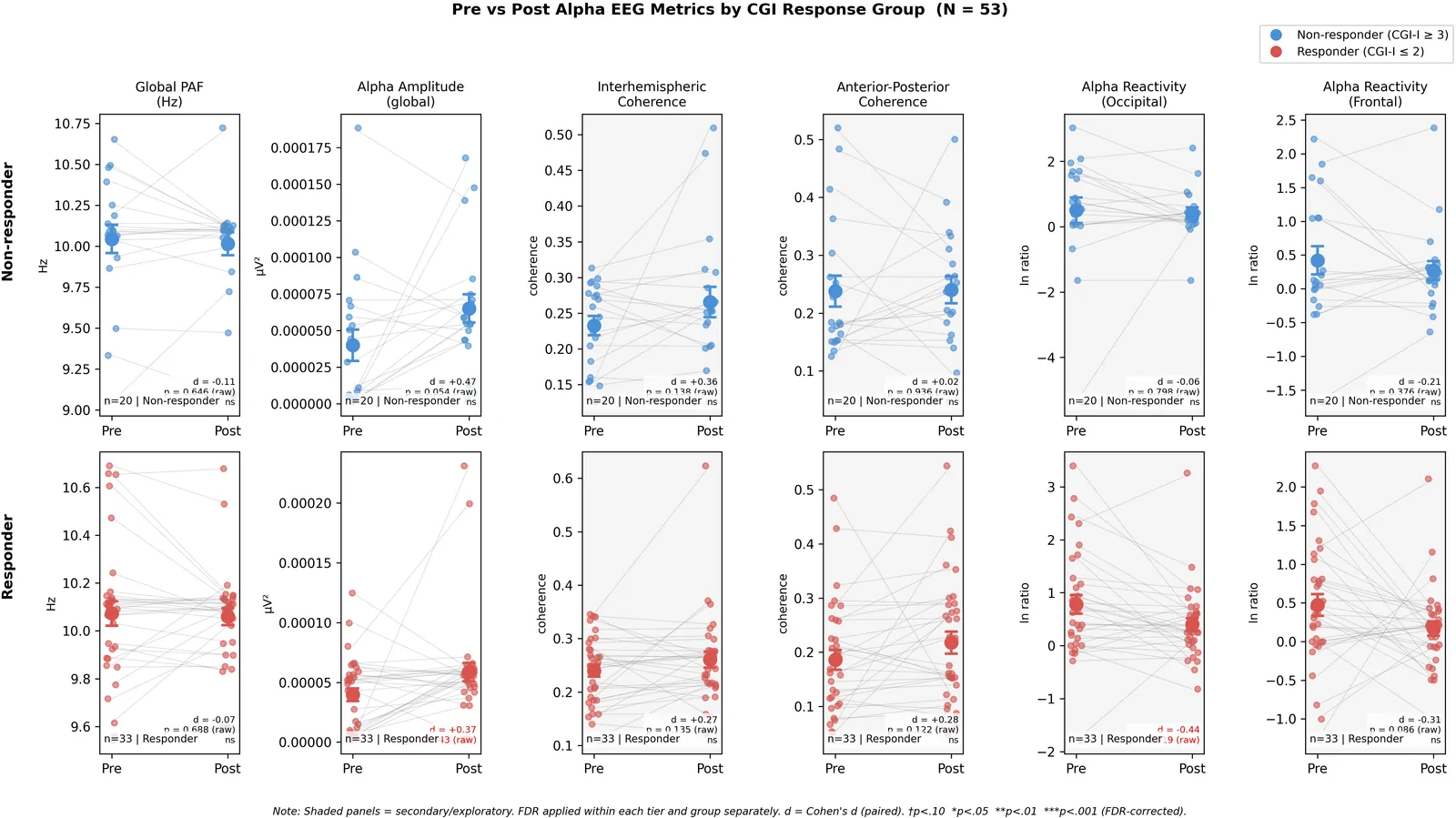
Pre-versus post-treatment alpha EEG metrics by CGI-I response group (N = 53). Top row: non-responders (CGI-I ≥ 3; n = 20); bottom row: responders (CGI-I ≤ 2; n = 33). Individual participant trajectories are shown by connecting lines, with group means and standard errors displayed. Cohen’s d represents the paired-samples effect size. Raw p-values are displayed within each panel, with FDR correction applied separately within each outcome tier and response group. Shaded panels indicate exploratory outcomes. †p <.10, *p <.05 (FDR-corrected).

#### 4.6.2 Treatment-related electrophysiological changes

Treatment-related electrophysiological changes were examined separately in responders and non-responders to explore whether patterns of change differed according to clinical outcome (**Figures 4**, **5**).

**Figure 5:**
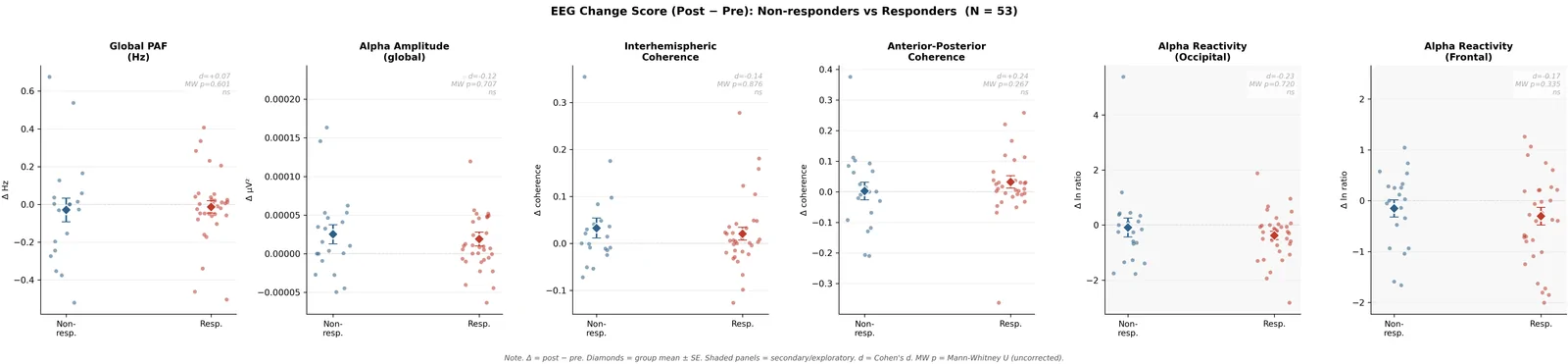
EEG change scores comparing non-responders (*n* = 20) and responders (*n* = 33). Individual data points represent participant-level change scores, with group means (diamonds) and standard errors displayed. Cohen’s *d* is reported as a descriptive estimate of the standardised between-group difference in change. Mann–Whitney *U* tests were used for between-group comparisons, with uncorrected *p*-values reported. Shaded panels indicate exploratory outcomes. PAF = peak alpha frequency.

For the primary electrophysiological outcomes, both groups showed increases in global alpha amplitude following neurofeedback. Responders exhibited a small-to-medium increase (d = 0.37, raw p = .013), whereas the corresponding increase amongst non-responders did not reach statistical significance (d = 0.47, raw p = .054). However, the magnitude of alpha-amplitude change did not differ significantly between responders and non-responders, and these within-group findings should therefore not be interpreted as evidence of a differential treatment effect between groups. No significant within-group changes were observed for peak alpha frequency or either coherence measure.

Amongst the secondary electrophysiological outcomes, responders demonstrated a significant reduction in occipital alpha reactivity, decreasing from 0.78 at baseline to 0.40 following treatment (d = −0.43, pFDR = .028). In contrast, occipital alpha reactivity remained essentially unchanged in non-responders (0.50 to 0.41; d = −0.06, pFDR = .348). Frontal alpha reactivity showed a similar, although non-significant, pattern amongst responders (d = −0.31, pFDR = .106).

Responders also showed a modest increase in anterior–posterior alpha coherence (d = 0.28, p = .048), whereas coherence remained essentially unchanged in non-responders (d = 0.02, p = .898). However, the increase in responders did not remain statistically significant following FDR correction.

Descriptively, eyes-open and eyes-closed alpha amplitudes showed somewhat different patterns of change between responders and non-responders (**Figure 3**). However, the direct responder versus non-responder comparison of the interaction contrast was not statistically significant (*t*(51) = 0.88, *p* = .381, *d* = 0.24). A Mann–Whitney sensitivity analysis was likewise non-significant (*U* = 350, *p* = .720). Thus, the observed patterns do not establish a statistically reliable difference in state-dependent alpha modulation between response groups.

### 4.7 Associations between electrophysiological and clinical change

Spearman correlations between changes in resting-state EEG measures and overall clinical improvement (CGI-I) are summarised in **Table 5**. No significant associations were observed between electrophysiological change and clinical improvement. Amongst the primary outcomes, the largest association was between change in anterior–posterior alpha coherence and CGI-I (ρ = 0.16, p = .267). Associations for global alpha amplitude (ρ = −0.05, p = .704), PAF (ρ = 0.07, p = .599), and interhemispheric coherence (ρ = −0.02, p = .871) were small and non-significant. Changes in occipital (ρ = −0.05, p = .717) and frontal alpha reactivity (ρ = −0.14, p = .336) were likewise not significantly associated with CGI-I.

**Table 5:** Spearman correlations between EEG change scores and clinical improvement (CGI-I).

EEG measure	*r*_s_	*p*	95% CI
Global PAF	.074	.599	[−.20, .34]
Global alpha amplitude	−.053	.704	[−.32, .22]
Interhemispheric coherence	−.023	.871	[−.29, .25]
Anterior-posterior coherence	.155	.267	[−.12, .41]
Occipital alpha reactivity	−.051	.717	[−.32, .22]
Frontal alpha reactivity	−.135	.336	[−.39, .14]

N = 53. CGI-I = Clinical Global Impression–Improvement. Lower CGI-I scores indicate greater clinical improvement. EEG change scores were calculated as post-treatment minus pre-treatment values. No correlations reached statistical significance (all *p* >.05).

## 5 Discussion

### 5.1 Principal findings

Approximately two-thirds of treatment-resistant adult refugees with persistent trauma-related symptoms were rated as clinically improved during multidisciplinary treatment incorporating individualised neurofeedback. Alongside these clinical changes, resting-state EEG demonstrated a selective rather than widespread pattern of electrophysiological change. Global alpha amplitude increased as hypothesised, whereas alpha reactivity changed in the opposite direction to our original expectation, and peak alpha frequency and alpha-band coherence remained stable following correction for multiple comparisons. Collectively, these findings suggest that complementary properties of resting-state alpha activity differ in their sensitivity to treatment-associated electrophysiological change.

Exploratory analyses suggested different patterns of eyes-open and eyes-closed alpha amplitude change in responders and non-responders. However, the direct comparison of the interaction contrast was not statistically significant. These findings therefore do not provide evidence of responder-specific differences in state-dependent alpha modulation and should be interpreted as descriptive. Changes in individual EEG measures were also not significantly associated with overall clinical improvement after correction for multiple comparisons.

Finally, quantitative analysis of more than 2,300 neurofeedback protocol applications demonstrated that treatment individualisation in routine specialist practice occurred primarily through progressive modification of electrode placement, whereas reinforcement frequencies remained comparatively stable within the alpha range. To our knowledge, this provides one of the first quantitative descriptions of how highly individualised neurofeedback is operationalised in routine clinical practice, suggesting that protocol adaptation was achieved predominantly through spatial targeting rather than frequent changes in reinforcement frequency. Beyond informing interpretation of the present findings, these observations contribute empirical evidence on the practical implementation of regulation-oriented neurofeedback, addressing a methodological aspect of the field that has rarely been quantified. The relative stability of reinforcement frequencies, despite modest upward and downward titration within the alpha range and substantial variation in electrode placement, raises the possibility that experienced clinicians individualise treatment primarily through spatial targeting whilst using reinforcement frequency adjustments to fine-tune treatment according to individual response. Whether the predominance of alpha-range reinforcement frequencies reflects a common regulatory window across diverse clinical presentations or simply current clinical practice remains uncertain and warrants further investigation. It will be important to interrogate this finding in the light of other clinical centre’s practice.

### 5.2 Clinical outcomes in a treatment-resistant, clinically heterogeneous cohort

The clinical significance of these findings should be interpreted in the context of the study population. Participants had been referred for neurofeedback because clinically significant trauma-related symptoms had persisted despite previous trauma-focused psychological treatment and, where appropriate, pharmacotherapy. In addition, the cohort reflected the complexity commonly encountered in specialist refugee mental health services, including extensive psychiatric comorbidity, psychotropic medication use, cumulative exposure to war, torture and forced displacement, and ongoing post-migration adversity.

Against this background, clinically meaningful improvement in 62.3% of participants indicates that substantial recovery remained possible despite prolonged illness and multiple barriers to treatment response. Although the present study was designed primarily to investigate neurophysiological changes associated with neurofeedback rather than treatment efficacy, these clinical outcomes are noteworthy given that participants had not responded adequately to previous interventions and presented with substantial psychiatric and psychosocial complexity. Refugees with chronic trauma-related psychopathology are frequently underrepresented in clinical trials despite representing a substantial proportion of clients seen within specialist trauma services. Importantly, participants rarely presented with a single, clearly defined disorder. Instead, they experienced multiple interacting trauma-related difficulties, including PTSD, depressive disorders, anxiety disorders, sleep disturbance, somatic symptoms and dissociative symptoms, reflecting the complex and overlapping clinical presentations increasingly recognised as characteristic of forcibly displaced populations rather than the exception (1–4, 7–9).

Baseline comparisons indicated that responders had higher educational attainment, were less likely to require interpreter services, and were more likely to report early childhood trauma than non-responders. These findings should be interpreted cautiously given the observational design and modest sample size. Nevertheless, they suggest that individual demographic, developmental, and communication-related factors may be associated with treatment response and warrant further investigation in larger prospective studies.

The coexistence of clinically meaningful improvement and selective electrophysiological changes supports the value of investigating neural regulation from a dimensional rather than diagnosis-specific perspective. This dimensional perspective is aligned with contemporary models that conceptualise chronic trauma as involving disturbances in interacting neural systems underlying self-regulation rather than dysfunction confined to individual diagnostic categories (2, 12). However, because diagnosis-specific and symptom-domain analyses were not undertaken, the present study cannot determine whether the observed electrophysiological changes reflect a shared transdiagnostic process or different pathways of change across participants.

Nevertheless, these findings should not be interpreted as evidence of the efficacy of neurofeedback alone. Participants received neurofeedback within an integrated multidisciplinary programme that also included trauma counselling, psychiatric management and psychosocial support, and the study did not include a comparison group. Furthermore, analyses were limited to participants who completed neurofeedback and both EEG assessments, potentially selecting individuals who were more able to engage with treatment. Consequently, the observed clinical improvement cannot be attributed specifically to neurofeedback.

### 5.3 Alpha oscillatory activity and state-dependent neural regulation

The present findings indicate selective changes across complementary properties of resting-state alpha oscillatory activity during multidisciplinary treatment incorporating neurofeedback. Global alpha amplitude increased following treatment, whereas alpha reactivity changed in the opposite direction to our original expectation, decreasing rather than increasing following neurofeedback. In contrast, peak alpha frequency and alpha-band coherence remained stable after correction for multiple comparisons. This pattern suggests that different measures of resting-state alpha activity may reflect distinct aspects of treatment-related electrophysiological change.

The observed increase in global alpha amplitude was consistent with our *a priori* hypothesis and extends previous evidence that neurofeedback may modify resting alpha oscillations. Contemporary models conceptualise the alpha rhythm as an active regulator of cortical excitability, influencing the processing of task-irrelevant information whilst supporting selective attention, sensory suppression, and resistance to distraction (14, 16, 60, 61). These functions are particularly relevant to trauma-related psychopathology, in which persistent hypervigilance, exaggerated threat monitoring, and altered salience processing are prominent clinical features (11, 13). Nicholson et al. (30) similarly reported increased alpha-band synchronisation following alpha-rhythm neurofeedback in individuals with PTSD, accompanied by altered amygdala connectivity and reductions in PTSD symptoms. Although their targeted, single-session intervention differed substantially from the prolonged, individualised regulation-oriented treatment examined here, the convergence of findings suggests that modulation of resting alpha activity may represent a common neurophysiological feature observed across different neurofeedback approaches. However, in the present study, increases in global alpha amplitude were observed in both clinically improved and non-improved participants, and changes in alpha amplitude were not significantly associated with clinical improvement. These findings suggest that enhanced resting alpha activity may reflect a broader physiological change occurring during treatment rather than a specific marker of symptomatic improvement. The absence of significant associations between conventional resting-state EEG measures and overall clinical improvement also suggests that changes in these measures should not be interpreted as direct electrophysiological markers of clinical response. At the same time, the differing patterns observed across alpha measures support examining state-dependent properties of alpha activity alongside absolute oscillatory amplitude.

The most unexpected finding concerned alpha reactivity. We originally hypothesised that neurofeedback would strengthen physiological alpha blocking, reflected by a greater difference between eyes-closed and eyes-open alpha activity. Instead, occipital alpha reactivity was significantly reduced following treatment. Descriptive examination indicated that alpha amplitude increased in both eyes-open and eyes-closed conditions, suggesting that the reduction in reactivity reflected a change in the relative modulation of alpha across recording states rather than a simple reduction in alpha activity. Exploratory subgroup analyses showed somewhat different patterns of eyes-open and eyes-closed alpha change in responders and non-responders; however, the direct between-group comparison was not statistically significant. These subgroup patterns should therefore be regarded as descriptive and do not provide evidence that changes in alpha reactivity differed according to clinical response.

One possible interpretation is that alpha reactivity reflects one aspect of a broader capacity for regulatory flexibility, conceptualised here as the ability to modulate cortical oscillatory activity across changing behavioural states and functional demands. From this perspective, alpha amplitude, alpha reactivity, and other dynamic properties of alpha oscillations may capture complementary aspects of cortical regulation rather than independent physiological phenomena. This interpretation is consistent with evidence suggesting that the functional significance of alpha oscillations lies not only in their absolute amplitude but also in their capacity for dynamic modulation. For example, Ros et al. (62) reported that neurofeedback restored the dynamic range of alpha oscillations following stroke, proposing that the capacity of alpha activity to fluctuate dynamically may be informative beyond static measures of alpha power alone. Although alpha variability and alpha reactivity quantify different electrophysiological properties, both may provide complementary information about the dynamic regulation of alpha activity.

The potential relevance of state-dependent alpha modulation is also supported by previous resting-state EEG studies. Contemporary resting-state EEG guidelines recommend including both eyes-open and eyes-closed recordings because they engage partially distinct neurophysiological processes related to vigilance, sensory processing, and resting brain function (63). In keeping with this distinction, Clancy et al. (18) reported reduced posterior alpha power together with reduced communication between posterior and frontal brain regions during eyes-open, but not eyes-closed, resting-state EEG in adults with PTSD, interpreting these findings as reflecting diminished cortical inhibition and increased sensory hyperresponsivity associated with hypervigilance. Similarly, Marcu et al. (21) observed reduced temporal-posterior alpha power during eyes-open, but not eyes-closed, resting-state EEG in adolescents exposed to complex childhood trauma. Although neither study examined longitudinal changes in alpha reactivity, both suggest that trauma-related alpha abnormalities may differ according to recording condition and, in these studies, were observed more prominently during eyes-open than eyes-closed conditions, supporting the relevance of examining eyes-open and eyes-closed alpha activity separately in trauma-exposed populations.

Within this framework, the descriptive subgroup pattern raises the possibility that eyes-open resting EEG may be sensitive to aspects of treatment-related electrophysiological change. Eyes-open and eyes-closed resting states differ in visual input and associated sensory and attentional demands, providing a rationale for examining them separately. However, the direct responder versus non-responder comparison of state-dependent alpha change was not statistically significant. The observed subgroup pattern therefore cannot establish greater eyes-open modulation amongst clinical responders, and its potential relevance to treatment response requires investigation in adequately powered prospective studies. This interpretation remains speculative but is consistent with contemporary models of alpha function and previous eyes-open findings in PTSD.

At the same time, changes in individual electrophysiological measures were not significantly associated with overall clinical improvement after correction for multiple comparisons. This is not unexpected, as the Clinical Global Impression reflects overall functioning across multiple symptom domains, including PTSD symptoms, mood, sleep, cognition, interpersonal functioning, and daily functioning, whereas alpha reactivity reflects a specific aspect of cortical physiology. Rather than functioning as a direct biomarker of overall clinical improvement, alpha reactivity may represent one component of the broader neurophysiological processes associated with treatment-related change.

Overall, the findings suggest selective changes across complementary properties of resting-state alpha activity, consistent with altered regulatory flexibility rather than uniform changes across all electrophysiological measures. Whilst global alpha amplitude increased following treatment, alpha reactivity changed in a different direction, highlighting the value of examining multiple dynamic indices of alpha function rather than relying on a single electrophysiological measure. Together, these findings suggest that dynamic measures reflecting the flexible modulation of cortical activity across behavioural states may provide greater insight into treatment-related neurophysiological change than absolute measures of oscillatory power alone. However, normative longitudinal data for alpha reactivity are currently lacking, making it difficult to determine whether the observed changes represent movement towards a normative physiological range. Although this interpretation requires confirmation in prospective controlled studies with longitudinal comparison groups, the present findings suggest that alpha reactivity may be a promising electrophysiological measure for investigating treatment-associated changes in neural regulation following neurofeedback in individuals with chronic trauma.

### 5.4 Stability of peak alpha frequency

Peak alpha frequency (PAF) remained stable following treatment and did not change significantly after correction for multiple comparisons. The absence of significant change is in line with the current understanding of PAF as a relatively stable neurophysiological characteristic associated with cognitive preparedness and cognitive efficiency, with theoretical models linking individual differences in PAF to the timing of thalamocortical feedback loops (22–24). PAF demonstrates high test–retest reliability, although modest within-person variation has been reported in association with cognitive task demands and other physiological influences. By comparison, alpha amplitude is particularly sensitive to changes in vigilance, sensory input, recording condition, and cognitive demands (23, 24, 64).

Although alterations in PAF have been reported in PTSD and pain-related conditions, evidence regarding treatment-related changes remains limited (25, 65). In a pilot study of individuals with comorbid PTSD and major depressive disorder, Petrosino et al. (66) reported no significant changes in individual alpha frequency following a course of repetitive transcranial magnetic stimulation, a finding consistent with the broader view of PAF as a relatively stable trait-like electrophysiological characteristic. The absence of detectable change in the present study similarly contrasts with the treatment-related changes observed in alpha amplitude and alpha reactivity, suggesting that PAF may reflect a comparatively stable aspect of alpha oscillatory dynamics over the treatment period examined. Whether PAF is modified by longer-term treatment or repeated neurofeedback remains an important question for future research.

Although the present study did not include a longitudinal healthy control group, PAF has consistently demonstrated good-to-excellent test–retest reliability in healthy adults across intervals ranging from weeks to several months, with some evidence of stability over longer periods (24, 67, 68). This suggests that the absence of significant change in PAF is unlikely to be explained solely by measurement instability. Nevertheless, without a longitudinal healthy control group, it remains impossible to determine whether the observed stability exceeded normal temporal variability or whether neurofeedback had no measurable effect on PAF.

### 5.5 Functional connectivity

Neither interhemispheric nor anterior–posterior alpha coherence changed significantly following correction for multiple comparisons, although both measures showed modest increases with small effect sizes. Scalp EEG coherence provides an indirect and spatially coarse measure of frequency-specific statistical coupling between signals recorded at different scalp locations and has been used to investigate large-scale patterns of cortical synchronisation. Its physiological interpretation is nevertheless influenced by factors including volume conduction, reference choice, spatial scale and the selected electrode pairings (50). The broader functional relevance of neuronal coherence is supported by theoretical models proposing that phase-aligned oscillatory activity facilitates communication between neuronal populations (26–28).

The absence of significant change in the present coherence measures does not exclude the possibility of connectivity changes that were not captured by the scalp-level metrics examined. Scalp EEG coherence cannot comprehensively localise or characterise distributed cortical and subcortical networks. Reflecting this limitation, functional neuroimaging studies have reported changes in default mode, salience and amygdala-centred connectivity following a single session of alpha-rhythm neurofeedback in individuals with PTSD (29, 30). These findings involved a different imaging modality, intervention duration and level of spatial analysis and therefore cannot be directly compared with the present results, but they illustrate that network-level changes may occur without being reflected in conventional scalp coherence measures.

The increase in global alpha amplitude in the absence of significant coherence change also indicates that alpha amplitude and long-range alpha coherence did not change in parallel. This is physiologically plausible because spectral amplitude and coherence represent related but distinct properties of oscillatory activity: amplitude reflects the magnitude of activity within a signal, whereas coherence reflects the consistency of frequency-specific relationships between signals. The present findings therefore suggest that treatment-associated changes were more readily detected in alpha amplitude and reactivity than in the interhemispheric and anterior–posterior coherence measures examined. Future studies using source-resolved EEG, alternative connectivity metrics and multimodal neuroimaging will be important for determining whether treatment-associated changes occur within distributed cortical or subcortical networks.

### 5.6 Contributions to the neurofeedback literature

This study extends previous neurofeedback research in several important respects. First, it provides a comprehensive evaluations of resting-state alpha electrophysiology following multidisciplinary treatment incorporating neurofeedback in a clinically complex cohort of refugees with persistent trauma-related symptoms receiving routine specialist care. Rather than focusing on a single electrophysiological outcome, the study simultaneously examined alpha amplitude, alpha reactivity, peak alpha frequency and alpha coherence, providing a more comprehensive assessment of resting-state alpha function than has typically been reported in neurofeedback research.

The study also provides one of the first quantitative characterisations of neurofeedback protocol implementation in clinical settings. Although treatment was highly individualised, analysis of more than 2,300 protocol applications demonstrated remarkably consistent reward frequencies, with 95.8% of reward-frequency ranges overlapping the alpha band and a session-weighted mean reward frequency of 11.63 Hz, whereas electrode placements varied substantially throughout treatment. This pattern suggests that treatment individualisation primarily occurred through adaptation of spatial targets whilst maintaining reward frequencies within the alpha range. These findings provide important clinical context for interpreting the electrophysiological results and represent one of the few comprehensive empirical descriptions of neurofeedback protocol implementation in routine specialist practice.

A further contribution of this study is the exploratory comparison of electrophysiological changes between clinically defined responders and non-responders. Although individual EEG measures were not significantly associated with overall clinical improvement, responder analyses identified descriptively different patterns of state-dependent alpha change that warrant further investigation, particularly in the eyes-open condition. Nevertheless, the findings highlight the potential value of incorporating clinically meaningful response categories alongside multidimensional electrophysiological assessment, rather than relying exclusively on whole-group averages, which may be less sensitive to heterogeneity in treatment-associated change.

### 5.7 Strengths, limitations and future directions

The principal strengths of this study lie in the combination of a large, clinically complex cohort, evaluation within routine specialist refugee mental health services, and the use of standardised resting-state EEG assessment under routine clinical conditions. Collectively, these features enhance the ecological validity of the findings and provide a detailed characterisation of clinical and electrophysiological changes observed during multidisciplinary treatment incorporating neurofeedback. From a methodological perspective, the study demonstrates that comprehensive resting-state EEG assessment can be implemented within specialist refugee mental health services using established procedures for EEG acquisition, preprocessing, and statistical analysis. Unlike highly controlled efficacy trials, the study was conducted under routine clinical conditions in a cohort characterised by extensive psychiatric comorbidity, severe trauma exposure, ongoing post-migration adversity, concurrent psychotropic medication use, and prolonged previous treatment.

These strengths should be considered alongside several limitations. Most importantly, the retrospective observational design and the absence of a control group preclude conclusions regarding treatment efficacy or causality. Participants received neurofeedback as one component of an integrated multidisciplinary programme that also included trauma counselling, psychiatric management, and psychosocial support. In addition, neurofeedback and counselling were delivered concurrently by the same clinicians. Consequently, the observed clinical and electrophysiological changes cannot be attributed specifically to neurofeedback and may instead reflect the combined effects of multiple interventions together with other unmeasured influences.

Clinical improvement was assessed using retrospectively derived CGI-I ratings rather than prospectively administered, validated symptom-specific or functional outcome measures. Although all records were independently assessed by two registered psychologists who had no knowledge of the pre–post EEG changes or the study hypotheses, raters necessarily had access to clinical progress documented during treatment, introducing potential assessment bias. The original independent ratings were not retained, preventing calculation of inter-rater reliability. The CGI-I provides a global measure of clinical change and does not identify which specific symptom or functional domains contributed to improvement.

The requirement for both baseline and post-treatment EEG resulted in exclusion of participants without complete longitudinal recordings and may have introduced selection bias. Participants who remained engaged sufficiently to complete follow-up EEG assessment may have differed from those without follow-up recordings in ways relevant to treatment engagement or outcome. Reasons for missing follow-up EEG were not systematically recorded, preventing evaluation of these potential differences. The analytical cohort should therefore not be assumed to represent all clients commencing neurofeedback within the service. Treatment duration varied considerably across participants, resulting in variation in the timing of post-treatment EEG assessment. However, sensitivity analyses restricted to the DONE 2 subgroup produced a pattern consistent with the full-sample analysis.

The highly individualised intervention reflects routine clinical practice but precludes conclusions regarding the relative contribution of individual protocol components, including electrode placements, protocol sequencing, and other treatment adaptations, to the observed outcomes. Similarly, although the Clinical Global Impression scale was well suited to this transdiagnostic cohort by capturing overall clinical improvement, it limited examination of relationships between electrophysiological change and specific functional domains. In addition, responders and non-responders differed on several baseline characteristics, including educational attainment, interpreter use, and childhood trauma exposure. Consequently, residual confounding cannot be excluded, and these baseline differences should be considered when interpreting comparisons between response groups. Finally, psychotropic medication use was common within the cohort, and changes in medication, physical health, or psychosocial circumstances during treatment may also have contributed to the observed clinical and electrophysiological changes.

Considerable inter-individual variability was observed across electrophysiological measures, particularly for alpha reactivity. Given the clinical complexity of this treatment-resistant refugee cohort, such variability was anticipated and likely reflects differences in trauma history, psychiatric comorbidity, medication use, and the highly individualised nature of the neurofeedback intervention. Consequently, modest group-level effects may have obscured meaningful individual patterns of neurophysiological change, and the absence of significant associations between electrophysiological and clinical outcomes should not be interpreted as evidence that neurophysiological changes are unrelated to recovery. Future studies using larger samples and repeated assessments may help to identify clinically meaningful response subgroups and sources of variability.

Although EEG assessments were generally conducted before 2:00 pm to minimise fatigue, the exact time of recording and participants’ sleep–wake state were not systematically matched between pre- and post-treatment assessments. Caffeine and nicotine consumption prior to recording was routinely documented but was not systematically restricted or examined in the present analysis. Participants were also receiving heterogeneous pharmacological treatment, and medication type, dose, timing and changes over the treatment period were not controlled analytically. These state-dependent and pharmacological factors may have introduced additional variability into the resting-state EEG measures. The sample size also limited sensitivity to detect smaller effects, particularly in subgroup analyses. Accordingly, null findings and exploratory subgroup comparisons should be interpreted cautiously.

Inspection of the change-score distributions identified several potential outliers. Review of these observations indicated that they were physiologically plausible rather than artefactual, and sensitivity analyses demonstrated that retaining them did not materially influence the results or their interpretation, supporting the robustness of the reported findings (**Supplementary Material S1**).

The present findings also highlight the limitations of relying exclusively on conventional resting-state spectral EEG measures. Although scalp EEG provides excellent temporal resolution, it cannot directly characterise activity within deep cortical or subcortical structures or quantify interactions within distributed large-scale brain networks. Furthermore, the analyses focused on alpha-band activity and selected coherence measures, capturing only a subset of the neurophysiological processes that may contribute to recovery following neurofeedback. Accordingly, the interpretations presented throughout this Discussion should be regarded as hypothesis-generating rather than mechanistic explanations of the observed electrophysiological changes.

Future prospective studies should incorporate independent outcome assessment together with validated measures of trauma-related symptoms, functioning, cognition and behavioural change. Controlled designs are needed to distinguish changes associated specifically with neurofeedback from those attributable to concurrent counselling, medication, psychosocial support, time, expectancy or other influences. Larger samples and prospectively standardised EEG assessment timepoints would also permit more rigorous evaluation of response subgroups and state-dependent alpha dynamics.

### 5.8 Conclusions

During routine multidisciplinary treatment incorporating individualised neurofeedback, approximately two-thirds of refugees with persistent trauma-related symptoms were rated as clinically improved. Because neurofeedback was delivered concurrently with trauma counselling and other clinical interventions, and because the study was retrospective and uncontrolled, these observations should not be interpreted as evidence that neurofeedback caused the clinical or electrophysiological changes.

The electrophysiological findings were selective: global alpha amplitude increased and occipital alpha reactivity decreased, whereas peak alpha frequency and alpha coherence remained stable following correction for multiple comparisons. This pattern suggests that absolute alpha amplitude and state-dependent alpha modulation may capture complementary aspects of treatment-associated electrophysiological change. Exploratory response-group analyses also identified descriptive differences in eyes-open and eyes-closed alpha dynamics, although these were not supported by significant direct between-group differences and require prospective investigation.

The quantitative analysis of more than 2,300 neurofeedback protocol applications further demonstrated that treatment individualisation occurred primarily through variation in electrode placement whilst reward frequencies remained concentrated within the alpha range, providing one of the first systematic empirical descriptions of neurofeedback protocol implementation in routine specialist clinical practice.

By integrating clinical outcomes, multidimensional resting-state EEG assessment, and quantitative characterisation of protocol implementation, this study provides a detailed characterisation of the electrophysiological changes associated with multidisciplinary treatment incorporating neurofeedback in complex trauma populations. The findings suggest that dynamic measures of alpha regulation, including eyes-open alpha activity and alpha reactivity, may provide greater insight into treatment-associated neurophysiological change than conventional measures of resting alpha amplitude alone. Future prospective controlled research incorporating multidimensional clinical outcomes, objective measures of cognitive and sensory regulation, and transdiagnostic frameworks will be important for determining whether these electrophysiological patterns are reproducible and whether dynamic aspects of alpha regulation provide more sensitive markers of treatment-associated neurophysiological change than conventional spectral EEG measures.

## Data Availability

The original contributions presented in the study are included in the article/**Supplementary Material**. Further inquiries can be directed to the corresponding author.
