# Supplementary material for: Clinical and resting-state EEG changes following neurofeedback in adult refugees with persistent trauma-related symptoms: a transdiagnostic pre–post observational study
Source: Front Psychiatry. 2026 Oct 7;17:1954165. doi: 10.3389/fpsyt.2026.1954165 (PMC13683770; doi:10.3389/fpsyt.2026.1954165)
Supplement: Supplementary file 1 [file Supplementaryfile1.docx]

**Supplementary Methods S1. Neurofeedback Protocol Analysis**

To characterise the neurofeedback intervention delivered during routine clinical care, neurofeedback protocol characteristics were analysed across all available neurofeedback treatment records from four treatment time points (T1–T4). Each REDCap treatment record permitted up to six protocol entries (nfb1–nfb6). For each protocol entry, electrode placement, number of protocol applications, and minimum and maximum reward frequencies were extracted.

Reward frequency was calculated as the midpoint of the documented minimum and maximum reward-frequency range and weighted by the corresponding number of protocol applications. Participant-level summaries describe the proportion of participants receiving each electrode placement at least once during treatment, whereas protocol-level summaries describe the frequency with which electrode placements and reward frequencies were applied across the treatment period. The resulting summaries are presented in Supplementary Tables S2–S5.
