# Supplementary material for: Clinical and resting-state EEG changes following neurofeedback in adult refugees with persistent trauma-related symptoms: a transdiagnostic pre–post observational study
Source: Front Psychiatry. 2026 Oct 7;17:1954165. doi: 10.3389/fpsyt.2026.1954165 (PMC13683770; doi:10.3389/fpsyt.2026.1954165)
Supplement: Supplementary file 2 [file Supplementaryfile2.docx]

# CRED-nf Checklist (Post Hoc Completion)

Manuscript title: Transdiagnostic Clinical and Resting-State Alpha Electrophysiological Changes Following Neurofeedback in Treatment-Resistant Adult Refugees: A Pre–Post Observational Study

Corresponding author: Mirjana Askovic

Email: mirjana.askovic@health.nsw.gov.au

## Section 1: Pre-experiment

| Item No. | Checklist Item | Manuscript Details |
| --- | --- | --- |
| 1a | Pre-register experimental protocol and planned analyses | Not preregistered; retrospective design. Addressed as a limitation. |
| 1b | Justify sample size | Sample size was determined by the number of participants with complete pre- and post-treatment resting-state EEG and clinical outcome data available for retrospective analysis. |

## Section 2: Control Groups

| Item No. | Checklist Item | Manuscript Details |
| --- | --- | --- |
| 2a | Employ control group(s) or control condition(s) | No control group; naturalistic pre–post observational study. |
| 2b | Double-blind design (when appropriate) | Not applicable. |
| 2c | Blind outcome raters and data analysts | Not blinded; acknowledged as a limitation. |
| 2d | Examine maintenance of blinding | Not applicable. |
| 2e | Use standard-of-care control in clinical efficacy trials | Not a clinical efficacy trial. |

## Section 3: Control Measures

| Item No. | Checklist Item | Manuscript Details |
| --- | --- | --- |
| 3a | Collect psychosocial factors | Demographic, trauma history and relevant clinical variables were collected and reported. |
| 3b | Report whether participants were provided with strategies | Neurofeedback protocols were individualised according to presenting symptoms, clinical assessment and therapist observations rather than standardised across participants. |
| 3c | Report participant-reported strategies | Not recorded. |
| 3d | Report online data processing and artefact correction | EEG preprocessing included visual inspection and artefact rejection procedures as described in the Methods. |
| 3e | Report condition/group effects for artefacts | Not assessed separately; artefact handling described in the Methods. |

## Section 4: Feedback Specifications

| Item No. | Checklist Item | Manuscript Details |
| --- | --- | --- |
| 4a | Define online feature extraction | Reward and inhibit frequency bands were individually selected according to clinical presentation and recorded using EEGer software. |
| 4b | Justify reinforcement schedule | Reinforcement threshold adjusted manually (target ~60–70% success). |
| 4c | Report feedback modality and content | Visual and auditory; e.g., “Formation” game used as feedback. |
| 4d | Report feedback variables or contrasts shown to participants | Inhibit/reward band amplitudes. |
| 4e | Report hardware/software | EEGer-4 software, J&J Spectrum 4-channel amplifier. Reported in Methods. |

## Section 5: Outcome Measures – Brain

| Item No. | Checklist Item | Manuscript Details |
| --- | --- | --- |
| 5a | Report neurofeedback regulation success | In-session neurofeedback regulation success was not quantified. |
| 5b | Plot within-/between-session feedback changes | Within-session neurofeedback learning curves were not recorded. |
| 5c | Compare experimental group to control group | No control group; analyses examined within-participant changes in resting-state EEG measures between pre- and post-treatment. |

## Section 6: Outcome Measures – Behaviour

| Item No. | Checklist Item | Manuscript Details |
| --- | --- | --- |
| 6a | Include a priori definitions of clinical/behavioural significance | Responders were defined a priori as participants rated as **very much improved** or **much improved** (CGI-I scores 1–2). Participants with CGI-I scores ≥3 were classified as non-responders. |
| 6b | Correlate regulation success and behavioural outcomes | Not applicable; neurofeedback regulation success was not quantified. |

## Section 7: Data Transparency

| Item No. | Checklist Item | Manuscript Details |
| --- | --- | --- |
| 7a | Upload materials, analysis code, and data to open repository | Not uploaded due to retrospective design and ethical/privacy considerations. Available upon reasonable request. |

Note: This CRED-nf checklist (Ros et al., 2020) has been completed post hoc to enhance transparency in reporting the neurofeedback components of the study. Deviations from checklist recommendations are acknowledged in the Limitations section.
