# Supplementary material for: Clinical and resting-state EEG changes following neurofeedback in adult refugees with persistent trauma-related symptoms: a transdiagnostic pre–post observational study
Source: Front Psychiatry. 2026 Oct 7;17:1954165. doi: 10.3389/fpsyt.2026.1954165 (PMC13683770; doi:10.3389/fpsyt.2026.1954165)
Supplement: Supplementary file 3 [file Supplementaryfile3.docx]

**Supplementary Figure S1. Distribution of midpoint reward frequencies across neurofeedback protocol applications.**

**Note:** Histogram illustrating the weighted distribution of midpoint reward frequencies across all frequency-complete neurofeedback protocol applications. Vertical dashed lines indicate the conventional alpha band (10–13 Hz). Reward frequencies were predominantly clustered within the alpha range, with 94.8% of protocol applications having reward-frequency ranges that overlapped 10–13 Hz.
