# Supplementary material for: Clinical and resting-state EEG changes following neurofeedback in adult refugees with persistent trauma-related symptoms: a transdiagnostic pre–post observational study
Source: Front Psychiatry. 2026 Oct 7;17:1954165. doi: 10.3389/fpsyt.2026.1954165 (PMC13683770; doi:10.3389/fpsyt.2026.1954165)
Supplement: Supplementary file 4 [file Supplementaryfile4.docx]

**Supplementary Figure S2. Reward-frequency distribution by electrode placement.**

Note: Heat map showing the percentage of protocol applications within each electrode placement falling into each midpoint reward-frequency category. Values were weighted by the number of protocol applications across all treatment time points (T1–T4).
