# Supplementary material for: Clinical and resting-state EEG changes following neurofeedback in adult refugees with persistent trauma-related symptoms: a transdiagnostic pre–post observational study
Source: Front Psychiatry. 2026 Oct 7;17:1954165. doi: 10.3389/fpsyt.2026.1954165 (PMC13683770; doi:10.3389/fpsyt.2026.1954165)
Supplement: Supplementary file 5 [file Supplementaryfile5.docx]

**Supplementary Material: Outlier Inspection and Sensitivity Analysis**

**Rationale.** To assess the robustness of the reported findings to influential observations, we inspected the distribution of pre- to post-treatment change scores (Δ = post − pre) for all six resting-state alpha EEG measures and conducted a sensitivity analysis in which potential outliers were excluded on a per-measure basis.

## **Outlier Identification**

Potential outliers were identified using standardised z-scores computed on the change-score distribution for each measure. Observations exceeding |z| > 2.5 were flagged for inspection. Across all six EEG measures, 10 observations from 8 unique participants met this criterion (Table S1, Figure S1). Most flagged observations were isolated to a single measure; only two participants (Subjects 7 and 15) were flagged on more than one measure.

The most extreme observation was Subject 29, a non-responder whose occipital alpha reactivity change score was 4.9 SD above the group mean (Δ = +5.38). This individual had exceptionally low baseline alpha amplitude (0.000011 μV², compared with the group mean of 0.000040 μV²), which rendered the log-ratio measure of alpha reactivity (ln[EC/EO]) unstable. Their baseline reactivity value of −5.37 was itself an extreme value, and the post-treatment value of +0.01 represented a return toward the group mean rather than an anomalous post-treatment response. This observation therefore reflects a statistical, rather than biological, outlier: an artefact of the log-ratio metric applied to very low-amplitude signals.

No flagged observation was attributable to data processing error, electrode artefact, or procedural irregularity. All flagged values were physiologically plausible and consistent with the substantial inter-individual variability characteristic of this treatment-resistant cohort.

***Table S1***

*Observations Exceeding |z| > 2.5 on EEG Change Score Distributions*

| **EEG Measure** | **Subject** | **Group** | ***Δ*** | ***z*** |
| --- | --- | --- | --- | --- |
| Global PAF | 25 | Non-resp. | +0.675 | 3.11 |
| Alpha amplitude | 7 | Resp. | +0.0002 | 4.00 |
| Alpha amplitude | 16 | Non-resp. | +0.0002 | 2.74 |
| IH coherence | 7 | Resp. | +0.278 | 3.05 |
| IH coherence | 15 | Non-resp. | +0.355 | 3.97 |
| AP coherence | 10 | Resp. | +0.357 | 2.80 |
| AP coherence | 15 | Non-resp. | +0.375 | 2.96 |
| AP coherence | 42 | Resp. | −0.363 | −3.20 |
| Occ alpha reactivity | 29 | Non-resp. | +5.382 | 4.92 |
| Fro alpha reactivity | 51 | Resp. | +2.434 | 3.01 |

*Note. Δ = post-treatment minus pre-treatment value. z = standardised score within each measure’s change-score distribution. Resp. = responder; Non-resp. = non-responder.*

## **Sensitivity Analysis**

To evaluate whether the flagged observations materially influenced the reported results, we repeated three sets of analyses after per-measure exclusion of observations with |z| > 2.5: (a) full-group within-subjects tests (Wilcoxon signed-rank) comparing pre- and post-treatment values, (b) between-group comparisons (Mann–Whitney U) of change scores between responders and non-responders, and (c) Spearman correlations between EEG change scores and clinical improvement (CGI-I). Results are summarised in Table S2 and Figure S2.

**Full-group pre–post comparisons.** The primary finding—a significant increase in global alpha amplitude—remained significant after exclusion of two outlying observations (Wilcoxon p = .004 → .011; d = 0.41 → 0.37). The secondary finding of reduced occipital alpha reactivity became *more* significant after exclusion (p = .006 → .002; d = −0.24 → −0.45), because the most extreme outlier (Subject 29) exhibited a large positive change that opposed the group-level trend toward reduced reactivity. The effect reported in the full sample is therefore conservative with respect to this observation. No non-significant result became significant, and no significant result became non-significant, for any primary outcome.

**Between-group comparisons.** All Mann–Whitney U comparisons between responders and non-responders on EEG change scores remained non-significant after outlier exclusion.

**CGI correlations.** All Spearman correlations between EEG change scores and clinical improvement remained non-significant after outlier exclusion.

**Conclusion.** No influential outliers were identified that would alter the interpretation of the reported findings. No flagged observation was attributable to artefact or data error. The primary and secondary conclusions of the study are robust to the exclusion of extreme observations. Given that the flagged values are consistent with genuine clinical heterogeneity in this treatment-resistant cohort, all observations are retained in the reported analyses.

***Table S2***

*Sensitivity Analysis: Full-Group Pre–Post Comparisons With and Without Outlier Exclusion*

| **EEG Measure** | ***n excl.*** | ***Full d*** | ***Full p*** | ***Excl. d*** | ***Excl. p*** | **Δ sig.** |
| --- | --- | --- | --- | --- | --- | --- |
| Global PAF | 1 | −0.09 | .383 | −0.16 | .255 | No |
| Alpha amplitude | 2 | +0.41 | .004** | +0.37 | .011* | No |
| IH coherence | 2 | +0.30 | .093† | +0.23 | .199 | No |
| AP coherence | 3 | +0.17 | .129 | +0.17 | .163 | No |
| Occ alpha reactivity | 1 | −0.24 | .006** | −0.45 | .002** | No |
| Fro alpha reactivity | 1 | −0.28 | .060† | −0.37 | .030* | No |

*Note. d = Cohen’s d (paired); p = Wilcoxon signed-rank test. Outliers defined as |z| > 2.5 on each measure’s change-score distribution. †p < .10, *p < .05, **p < .01. Δ sig. = whether the significance classification changed after exclusion.*

***Figure S1***

*Distribution of EEG change scores with outlier identification*

*Note. Each panel shows the distribution of change scores (Δ = post − pre) for one EEG measure. Responders are shown in red, non-responders in dark blue. Diamond markers indicate observations exceeding |z| > 2.5. Dashed orange lines indicate 1.5 × IQR fences. Grey horizontal line indicates the median.*

***Figure S2***

*Sensitivity analysis: effect of outlier exclusion on full-group change scores*

*Note. Bars show |Cohen’s d| for each EEG measure in the full sample (dark) and after per-measure exclusion of observations with |z| > 2.5 (red). Wilcoxon signed-rank p-values and significance labels are shown above each bar. †p < .10, *p < .05, **p < .01.*
