# Supplementary material for: Clinical and resting-state EEG changes following neurofeedback in adult refugees with persistent trauma-related symptoms: a transdiagnostic pre–post observational study
Source: Front Psychiatry. 2026 Oct 7;17:1954165. doi: 10.3389/fpsyt.2026.1954165 (PMC13683770; doi:10.3389/fpsyt.2026.1954165)
Supplement: Supplementary file 6 [file Table1.docx]

**Supplementary Table S1. Clinical Global Impression (CGI) Rating Criteria Used in the Study**

| **CGI measure** | **Score** | **Definition used in the study** |
| --- | --- | --- |
| **CGI-Severity (CGI-S)** | 1 | Normal, not at all ill |
|  | 2 | Borderline mentally ill |
|  | 3 | Mildly ill |
|  | 4 | Moderately ill |
|  | 5 | Markedly ill |
|  | 6 | Severely ill |
|  | 7 | Among the most extremely ill patients |
| **CGI-Improvement (CGI-I)** | 1 | Very much improved |
|  | 2 | Much improved |
|  | 3 | Minimally improved |
|  | 4 | No change |
|  | 5 | Minimally worse |
|  | 6 | Much worse |
|  | 7 | Very much worse |

**Note.** CGI ratings were completed independently by two registered psychologists using all available clinical information, including clinical interviews, progress notes, behavioural observations, standardised questionnaires, and psychiatric and psychological reports. Disagreements were resolved through consensus with a third senior clinician. Participants with **CGI-I scores of 1–2** were classified as responders, whereas participants with **CGI-I scores of 3–7** were classified as non-responders.
