# Supplementary material for: Clinical and resting-state EEG changes following neurofeedback in adult refugees with persistent trauma-related symptoms: a transdiagnostic pre–post observational study
Source: Front Psychiatry. 2026 Oct 7;17:1954165. doi: 10.3389/fpsyt.2026.1954165 (PMC13683770; doi:10.3389/fpsyt.2026.1954165)
Supplement: Supplementary file 7 [file Table2.docx]

**Supplementary Table S2. Summary of Neurofeedback Protocol Dataset**

| **Audit item** | **Value** |
| --- | --- |
| Participants included | 53 |
| Treatment records analysed (T1–T4) | 210 |
| Total recorded protocol applications | 2,587 |
| Standard coded electrode-placement applications | 2,361 |
| Free-text/individualised protocol applications | 226 |
| Frequency-complete protocol applications | 2,577 |
| Protocol applications with missing reward-frequency information | 10 |
| Overall weighted mean reward frequency (all protocols) | 11.58 Hz |
| Overall weighted mean reward frequency (standard coded placements) | 11.63 Hz |
| Reward-frequency ranges overlapping 10–13 Hz (all protocols) | 94.8% |
| Reward-frequency ranges overlapping 10–13 Hz (standard coded placements) | 95.8% |

**Note.** The main manuscript reports analyses based on the 2,361 standard coded electrode-placement applications. When free-text or other individualised placements are included, the complete dataset comprises 2,587 protocol applications.
