# Supplementary material for: Clinical and resting-state EEG changes following neurofeedback in adult refugees with persistent trauma-related symptoms: a transdiagnostic pre–post observational study
Source: Front Psychiatry. 2026 Oct 7;17:1954165. doi: 10.3389/fpsyt.2026.1954165 (PMC13683770; doi:10.3389/fpsyt.2026.1954165)
Supplement: Supplementary file 8 [file Table3.docx]

**Supplementary Table S3. Distribution of Midpoint Reward Frequencies Across Neurofeedback Protocol Applications**

| Midpoint reward-frequency category (Hz) | Protocol applications (n) | Percentage (%) |
| --- | --- | --- |
| <8.0 | 132 | 5.1 |
| 8.0–9.9 | 359 | 13.9 |
| 10.0–10.9 | 368 | 14.3 |
| 11.0–11.9 | 433 | 16.8 |
| 12.0–12.9 | 397 | 15.4 |
| 13.0–13.9 | 768 | 29.8 |
| ≥14.0 | 120 | 4.7 |
| Total | 2577 | 100.0 |

**Note.** Midpoint reward frequencies were calculated as the midpoint of the documented minimum and maximum reward-frequency range for each protocol entry and weighted by the corresponding number of protocol applications. The denominator comprises 2,577 protocol applications with complete reward-frequency information across all treatment time points (T1–T4). Ten protocol applications with incomplete reward-frequency data were excluded. Overall, 94.8% of reward-frequency ranges overlapped the 10–13 Hz alpha band.
