# Supplementary material for: Clinical and resting-state EEG changes following neurofeedback in adult refugees with persistent trauma-related symptoms: a transdiagnostic pre–post observational study
Source: Front Psychiatry. 2026 Oct 7;17:1954165. doi: 10.3389/fpsyt.2026.1954165 (PMC13683770; doi:10.3389/fpsyt.2026.1954165)
Supplement: Supplementary file 9 [file Table4.docx]

**Supplementary Table S4. Reward-Frequency Distribution by Electrode Placement**

| **Placement** | **n** | **Mean** | **SD** | **<8.0 %** | **8.0–9.9 %** | **10.0–10.9 %** | **11.0–11.9 %** | **12.0–12.9 %** | **13.0–13.9 %** | **≥14.0 %** |
| --- | --- | --- | --- | --- | --- | --- | --- | --- | --- | --- |
| T3–T4 | 712 | 11.71 | 1.42 | 2.2 | 7.3 | 9.6 | 36.7 | 21.1 | 23.2 | 0.0 |
| T4–P4 | 513 | 10.09 | 1.65 | 10.9 | 30.0 | 29.8 | 10.5 | 18.3 | 0.4 | 0.0 |
| Cz–A2 | 251 | 13.27 | 0.83 | 0.0 | 0.0 | 4.4 | 1.2 | 0.4 | 79.7 | 14.3 |
| Other individualised placements | 226 | 11.07 | 2.90 | 12.8 | 27.4 | 12.4 | 2.2 | 2.7 | 30.1 | 12.4 |
| C3–C4 | 214 | 12.54 | 1.48 | 0.0 | 15.4 | 8.9 | 2.8 | 6.1 | 66.8 | 0.0 |
| Cz–T6 | 174 | 13.23 | 0.86 | 0.0 | 0.0 | 0.0 | 10.9 | 9.8 | 55.2 | 24.1 |
| T5–T6 | 139 | 11.19 | 1.14 | 2.2 | 12.2 | 10.8 | 49.6 | 25.2 | 0.0 | 0.0 |
| Pz–A2 | 111 | 9.82 | 0.51 | 0.0 | 29.7 | 66.7 | 2.7 | 0.9 | 0.0 | 0.0 |
| C4–Pz | 68 | 11.79 | 2.20 | 8.8 | 10.3 | 0.0 | 0.0 | 61.8 | 19.1 | 0.0 |
| Fz–Pz | 55 | 13.35 | 0.93 | 0.0 | 0.0 | 0.0 | 0.0 | 38.2 | 36.4 | 25.5 |
| C4–A2 | 49 | 11.96 | 2.65 | 12.2 | 0.0 | 0.0 | 0.0 | 24.5 | 63.3 | 0.0 |
| Cz–T5 | 33 | 13.05 | 0.98 | 0.0 | 0.0 | 0.0 | 18.2 | 0.0 | 81.8 | 0.0 |
| C4–P4 | 19 | 11.15 | 1.90 | 15.8 | 5.3 | 0.0 | 36.8 | 26.3 | 15.8 | 0.0 |
| Iz–A2 | 13 | 7.08 | 0.19 | 100.0 | 0.0 | 0.0 | 0.0 | 0.0 | 0.0 | 0.0 |

**Note.** Reward frequencies represent the midpoint of the documented minimum and maximum reward-frequency range for each protocol entry and were weighted by the corresponding number of protocol applications across all treatment time points (T1–T4). Values in the frequency-category columns represent the percentage of protocol applications within each electrode placement. Reward-frequency distributions differed significantly across electrode placements (Kruskal–Wallis *H* = 1066.58, *p* < .001).
