# Supplementary material for: Clinical and resting-state EEG changes following neurofeedback in adult refugees with persistent trauma-related symptoms: a transdiagnostic pre–post observational study
Source: Front Psychiatry. 2026 Oct 7;17:1954165. doi: 10.3389/fpsyt.2026.1954165 (PMC13683770; doi:10.3389/fpsyt.2026.1954165)
Supplement: Supplementary file 10 [file Table5.docx]

**Supplementary Table S5. Active Neurofeedback Training Duration According to Electrode Placement**

| Electrode placement | Treatment records (n) | Mean duration (min) | Median (min) | Range (min) |
| --- | --- | --- | --- | --- |
| T3–T4 | 52 | 13.4 | 13.0 | 6.5–22.0 |
| T4–P4 | 46 | 13.4 | 13.0 | 7.0–22.0 |
| T5–T6 | 17 | 14.4 | 14.0 | 10.0–19.0 |
| C3–C4 | 28 | 13.3 | 13.0 | 7.0–23.0 |
| C4–A2 | 12 | 13.3 | 12.5 | 9.0–22.0 |
| C4–P4 | 7 | 14.9 | 15.0 | 10.0–22.0 |
| C4–Pz | 8 | 13.5 | 13.0 | 10.0–18.0 |
| Cz–A2 | 29 | 12.8 | 12.0 | 7.0–22.0 |
| Cz–T5 | 5 | 14.0 | 14.0 | 11.0–17.0 |
| Cz–T6 | 13 | 13.4 | 13.0 | 11.0–18.0 |
| Pz–A2 | 10 | 16.2 | 16.0 | 10.0–23.0 |
| Iz–A2 | 1 | 10.0 | 10.0 | 10.0–10.0 |
| Fz–Pz | 8 | 14.0 | 13.5 | 12.0–17.0 |

**Note.** Session duration data were available for a subset of treatment sessions in which active neurofeedback training time was documented. Values represent the duration of active neurofeedback training during sessions in which the corresponding electrode placement was used. These data are presented descriptively to illustrate typical training duration and should not be interpreted as representing all treatment sessions. Across electrode placements, active neurofeedback training typically lasted approximately **13–16 minutes per protocol**.
